# Tracking aid for global health goals: a systematic comparison of four approaches applied to reproductive, maternal, newborn, and child health

**DOI:** 10.1016/S2214-109X(18)30276-6

**Published:** 2018-07-14

**Authors:** Catherine Pitt, Christopher Grollman, Melisa Martinez-Alvarez, Leonardo Arregoces, Josephine Borghi

**Affiliations:** a Department of Global Health and Development, London School of Hygiene & Tropical Medicine, London, UK

## Abstract

**Background:**

Four initiatives have estimated the value of aid for reproductive, maternal, newborn, and child health (RMNCH): Countdown to 2015, the Institute for Health Metrics and Evaluation (IHME), the Muskoka Initiative, and the Organisation for Economic Co-operation and Development (OECD) policy marker. We aimed to compare the estimates, trends, and methodologies of these initiatives and make recommendations for future aid tracking.

**Methods:**

We compared estimates of aid for RMNCH from the four initiatives for all years available at the time of our analysis (1990–2016). We used publicly available datasets for IHME and Countdown. We produced estimates for Muskoka and the OECD policy marker using data in the OECD Creditor Reporting System. We sought to explain differences in estimates by critically comparing the methods used by each approach to identify and analyse aid, and quantifying the effects of these choices on estimates.

**Findings:**

All four approaches indicated substantial increases over time in global aid for RMNCH, but estimates of aid amounts and year-on-year trends differed substantially, especially for individual donors and recipient countries. Muskoka (US$ 13·0 billion in 2013, constant 2015 US$) and Countdown's RMNCH estimates ($13·1 billion in 2013) tended to be the highest and most similar, although they often indicated different year-on-year trends. IHME produced lower estimates ($10·8 billion in 2013), which often indicated different trends from the other approaches. The OECD policy marker produced by far the lowest estimates ($2·0 billion in 2013) because half of bilateral donors did not report on it consistently and those who did tended to apply it narrowly. Estimates differed across approaches primarily because of differences in methods for distinguishing aid for RMNCH from aid for other purposes; adjustments for inflation, exchange rates, and under-reporting; whether donors were credited for their support to multilateral institutions; and the handling of aid to unspecified recipients.

**Interpretation:**

The four approaches are likely to lead to different conclusions about whether individual donors and recipient countries have fulfilled their obligations and commitments and whether aid was sufficient, targeted to countries with greater need, or effective. We recommend that efforts to track aid for the Sustainable Development Goals reflect their multisectoral and interconnected nature and make analytical choices that are appropriate to their objectives, recognising the trade-offs between simplicity, timeliness, precision, accuracy, efficiency, flexibility, replicability, and the incentives that different metrics create for donors.

**Funding:**

Subgrant OPP1058954 from the US Fund for UNICEF under their Countdown to 2015 for Maternal, Newborn and Child Survival Grant from the Bill & Melinda Gates Foundation.

## Introduction

Estimating how much aid donors give to low-income and middle-income countries is essential, but complex. Aid estimates are required to hold donors and recipients accountable and to assess whether aid is sufficient, targeted to need, and effective. The Organisation for Economic Co-operation and Development (OECD) provides a standardised framework for donor aid reporting—the Creditor Reporting System—which facilitates estimates of total annual aid flows from most donors. However, to evaluate aid flows for specific health priorities, total aid flows need to be disaggregated.^1^ This disaggregation can be challenging because the categories required for evaluation do not always align with the ways in which funds are disbursed or reported.

Many resource tracking exercises have estimated the value of aid for specific diseases, including malaria,^2^, ^3^ tuberculosis,^4^, ^5^ pneumonia and diarrhoea,^6^ and HIV;^7^, ^8^ for neglected tropical diseases^9^, ^10^ and other groups of diseases;^11^, ^12^, ^13^ for mental health,^14^, ^15^ newborn health,^16^, ^17^ and reproductive health.^18^, ^19^, ^20^, ^21^ Some resource tracking exercises have assessed the distribution of aid across health areas.^13^, ^22^, ^23^, ^24^ Four initiatives have estimated the value of aid for reproductive, maternal, newborn, and child health (RMNCH): the Countdown to 2015;^25^, ^26^, ^27^, ^28^, ^29^, ^30^ the G8's Muskoka methodology,^31^ implemented by the Partnership for Maternal, Newborn & Child Health (PMNCH);^1^ the Institute for Health Metrics and Evaluation (IHME);^23^, ^32^ and the RMNCH policy marker within the OECD's Creditor Reporting System database (panel).^40^ Previous studies have included comparisons of the first three of these RMNCH initiatives and highlighted some of the differences in estimates and underlying methodologies.^1^, ^25^, ^30^ However, to our knowledge, no study to date has systematically compared estimated trends and amounts of aid for RMNCH across these four approaches or examined their methodologies in detail. This under-investigation represents an important research gap because different estimates of aid for RMNCH could lead to substantially different conclusions about past aid efforts, which could in turn affect future aid policies and health outcomes. Policy makers, advocates, and academics therefore require informed guidance regarding the relative merits of the different approaches, how to interpret their estimates, and how best to track aid for new global health goals.

Panel **Origins of the Creditor Reporting System purpose codes and four approaches for tracking aid for RMNCH**

The OECD's purpose codes have been used since the Creditor Reporting System aid activity database was established in 1973. The Creditor Reporting System provides a mechanism for comparable, detailed aid tracking as a complement to the aggregate statistics and sector codes in the OECD Development Assistance Committee database, which have been used since 1960. The Creditor Reporting System requires donors to categorise their funding records by the economic or social sector of activity, and within each sector, into a single purpose code. Although some sector and purpose codes have been added or modified over the years, the overall structure has remained unchanged. The number of donors reporting to the Creditor Reporting System has increased greatly from the 15 rich-country bilateral members of the OECD's Development Assistance Committee and five development banks reporting in 1973 to 50 bilateral, 35 multilateral, and one private donor reporting in 2017. The quality and completeness of data have also improved substantially; from 2003 onwards, the Creditor Reporting System contains detailed disbursement data for more than 90% of the aggregate data reported in the OECD Development Assistance Committee database.^33^ Data are initially reported in the Creditor Reporting System with a 12-month delay following the close of the calendar year of the disbursement and revised estimates are reported quarterly thereafter.

The Countdown to 2015 was established in 2005 as a multidisciplinary, multi-institutional collaboration to monitor and promote progress in the countries with the worst RMNCH outcomes for health outcomes, coverage of key interventions, and determinants of coverage, including financing. As the Creditor Reporting System purpose and sector codes were not structured to provide data on the value of aid supporting specific objectives, the London School of Hygiene & Tropical Medicine developed methods initially to track the value of funding promoting child health (MDG4), and subsequently expanded to include maternal and reproductive health. Estimates in total, by donor, and by recipient country (in aggregate and per relevant population) were published within the Countdown reports in 2008, 2011, 2013, 2015, and 2016 and as research articles on aid for MNCH in 2006,^29^ 2008,^26^ 2010,^28^ and 2012,^27^ on reproductive health in 2013,^18^ and on RMNCH in 2015^25^ and 2016.^30^ Analyses assessed funding from 2003, the earliest year for which relatively complete disbursement data were available, to 2013. This initiative continues as the Countdown to 2030, with an expanded remit, including adolescents and an explicit emphasis on nutrition. Methods for tracking donor financing in this next phase are under review.

The IHME was established at the University of Washington in 2007 with the aim to provide “an impartial, evidence-based picture of global health trends to inform the work of policymakers, researchers, and funders.”^34^ In 2009, IHME published the first in a series of annual Financing Global Health reports.^35^ Although focused on characterising overall aid flows for the health sector since 1990, this report also included estimates of the value of aid specifically for HIV, malaria, tuberculosis (MDG6), and health sector support to examine “whether the distribution of global health resources across different disease areas and geographical areas reflect current global health priorities… [and] the relationship of DAH [development assistance for health] to disease burden.”^35^ From 2010, additional health areas, notably MNCH, were examined in a series of research articles and in each annual report, the latest of which estimated aid through 2017.^24^, ^36^ We analysed the findings^32^ and methods^37^ used in IHME's 2016 report, which was available at the time of our analysis.

The Muskoka methodology was launched at the G8 summit in Muskoka, Canada in 2010, as a mutually agreed approach for G8 countries to monitor their own financial support for MDGs 4 and 5. The G8's health working group sought an approach that would be fully transparent, straightforward to implement, and accepted by donors; account for G8 members' core contributions to multilaterals and global health initiatives; and appropriately reflect the range of sectors that promote RMNCH. To this end, they consulted with the OECD, the London School of Hygiene & Tropical Medicine, and others, and developed methods, which they published online.^31^ The PMNCH then used the Muskoka methods (with a few modifications) to track disbursements in fulfilment of commitments to the Global Strategy for Women's and Children's Health (2010–15) in their annual accountability reports. The G8 set 2007 as the baseline against which to measure their subsequent disbursements, PMNCH reported on 2006–14, and we generated estimates using PMNCH's latest methods for estimating RMNCH disbursements for 2002–15.

The OECD's RMNCH policy marker was introduced on a trial basis in 2014 for reporting on flows from 2013 onwards within the Creditor Reporting System, following recommendations of the Commission on Information and Accountability for Women's and Children's Health and the Muskoka Initiative.^38^ Each policy marker in the Creditor Reporting System is a single, additional variable that indicates the degree to which the donor believes its disbursement supports a given cross-sectoral policy area (eg, the environment).^39^ Donors are expected to code each policy marker in addition to the purpose code for each disbursement record. In 2016, the OECD decided to fully adopt the RMNCH policy marker, although its specific coding framework remains under review.

RMNCH=reproductive, maternal, newborn, and child health. OECD=Organisation for Economic Co-operation and Development. MDG=Millenium Development Goal. MNCH=maternal, newborn, and child health. IHME=Institute for Health Metrics and Evaluation. PMNCH=Partnership for Maternal, Newborn, and Child Health.

**Research in context**

**Evidence before this study**

Four initiatives produced estimates of the value of aid for reproductive, maternal, newborn, and child health (RMNCH) over time, but to our knowledge no studies have systematically compared their estimates or methodologies in detail. Policy makers and academics thus lack informed guidance regarding the relative merits of the different approaches, how to interpret their estimates, and how best to track aid for the Sustainable Development Goals (SDGs) and the Global Strategy for Women's, Children's, and Adolescents' Health.

**Added value of this study**

Our in-depth comparison showed that the four RMNCH tracking initiatives produced substantially different estimates of levels and trends in aid for RMNCH, especially for individual donors and recipient countries. We explored how methodological differences led to these differing estimates and trends and found that differences both in the underlying conceptual frameworks and in many technical choices substantially influenced estimates. At the conceptual level, we showed that the approaches measured fundamentally different constructs. Muskoka, Countdown, and the Organisation for Economic Cooperation and Development (OECD) RMNCH policy marker sought to estimate the value of aid supporting RMNCH, whereas the Institute for Health Metrics and Evaluation (IHME) sought to characterise health sector aid by focus area. IHME estimates of aid for RMNCH thus reflect the value of aid explicitly described as promoting RMNCH, excluding aid oriented towards diseases or the humanitarian sector and most health systems funding, even where such aid would directly promote RMNCH.

We critically compared the technical choices of each approach and the degree to which different choices influenced estimates. As half of major donors have not implemented the OECD RMNCH policy marker, its estimates are low and uninformative. Although Countdown provided the most in-depth approach for identifying aid for RMNCH, it was slow and labour-intensive, making it not practically replicable, and not readily adaptable to new goals. The Muskoka approach was transparent, quick to implement, fully replicable, and readily adaptable to new goals, but carried a greater risk of misclassifying funding because it used high-level assumptions without project-level review. IHME's wider range of data sources led to substantial complexity in data management techniques, which were not fully replicable, but its techniques for apportioning aid to RMNCH were quick to implement, adaptable to new goals, largely replicable, and precise. By making substantial adjustments to donors' reported data, IHME generated estimates over a much longer time period than the other approaches. All four approaches involved many assumptions and none included funding for activities in education, transport, or social protection; quantified the uncertainty in their estimates; or included bilateral aid from China, Russia, India, or Brazil.

**Implications of all the available evidence**

The four approaches are likely to lead to different conclusions about whether individual donors and recipient countries have fulfilled their obligations and commitments and whether aid was sufficient, targeted to countries with greater need, or effective. To monitor the flow of funds supporting each SDG and the Global Strategy, methods for tracking aid should be built on a sound conceptual framework that recognises that funding can simultaneously support multiple objectives, even if these objectives are not named explicitly in reports of that funding. We recommend that future aid tracking efforts begin by identifying an explicit conceptual framework and then make technical choices appropriate to their objectives, recognising the trade-offs between simplicity, timeliness, precision, accuracy, efficiency, flexibility, and replicability. For accountability exercises, experts and stakeholders should consider refining the Muskoka approach to improve its precision and accuracy, while maintaining its simplicity and replicability. For in-depth analyses addressing specific research questions, combining key term searches and use of the OECD purpose codes with manual review of a restricted set of records could provide a more suitable balance between rigour and efficiency.

In our study, we aimed to compare the estimates and trends generated by the four RMNCH aid tracking initiatives—including Countdown, with which we were involved—and examine how their underlying methodologies affected estimates and trends. We aimed to inform future efforts to monitor resource flows, notably for the Global Strategy for Women's, Children's, and Adolescents' Health (2016–2030) and the Sustainable Development Goals (SDGs).

## Methods

### Comparison of estimates

To compare RMNCH aid disbursement levels and trends, we used the most recent publicly available datasets for IHME^41^ and Countdown^42^ at the time of our analysis and used the June, 2017, version of the Creditor Reporting System to produce estimates for the Muskoka method^31^ and the OECD policy marker.^40^ Previously published estimates^1^ for the Muskoka method did not present data for all recipient countries or annual data by donor and recipient country. To our knowledge, OECD policy marker estimates have not previously been published. We are not aware of other methods to generate estimates of aid for RMNCH as a whole.

We presented five metrics: Countdown's estimates of RMNCH and MNCH, Muskoka's RMNCH estimate, the RMNCH policy marker, and IHME's MNCH estimate (panel). We refer to these as estimates of aid for RMNCH, although each approach defined its metric(s) in different ways. In particular, Countdown created separate RMNCH and MNCH categories, while IHME defined its MNCH category to include both reproductive health and family planning. The initiatives reported on aid for RMNCH over different but overlapping time periods (panel), so we present all reported years but only compared aid levels and trends for years reported on by more than one initiative.

We used line graphs to compare estimates and trends in aid for RMNCH for all countries and for the 75 Countdown to 2015 priority recipients (based on health need; appendix). We also compared estimates by individual donor for 24 donors (longstanding members of the OECD's Development Assistance Committee, including the European Union [EU]) and by individual recipient for 24 recipients (the nine countries with the worst levels in 2013 for each of five indicators:^43^ maternal mortality ratio, number of maternal deaths, mortality in children younger than 5 years, number of deaths in children under 5 years, and female life expectancy). We explored whether the choice of aid tracking approach would affect conclusions about the association between aid and mortality using scatter plots of aid for RMNCH per child under 5 years^44^ compared with the under-5 mortality rate for each of the 75 priority countries using data for 2013. Estimates are presented in constant 2015 US$.

### Comparison of methods

We did an in-depth comparison of the methods underpinning each aid tracking initiative to explain similarities and differences in resulting aid estimates. We first compared the objectives of the different initiatives. We then examined how each initiative identified and analysed aid in general and how each distinguished aid for RMNCH from other aid. As the Muskoka, IHME, and Countdown approaches have evolved over time, we focused on their latest methods at the time of our analysis.

To better understand how the differences in methods affected estimates of aid for RMNCH, we replicated the IHME and Muskoka methods for distinguishing aid for RMNCH from other types of aid in the Countdown dataset,^42^ and restricted to the EU and 23 donor countries whose aid the IHME assessed in the Creditor Reporting System. We also compared the policy marker and the Muskoka methods in the June, 2017, Creditor Reporting System. We produced Sankey diagrams in SankeyMATIC (BETA) and line graphs in Microsoft Excel 2016 to illustrate how each method classified the same funding flows.

### Role of the funding source

The funder of the study had no role in study design, data collection, data analysis, data interpretation, or writing of the report. The corresponding author had full access to all the data in the study and had final responsibility for the decision to submit for publication.

## Results

**Figure 1:**
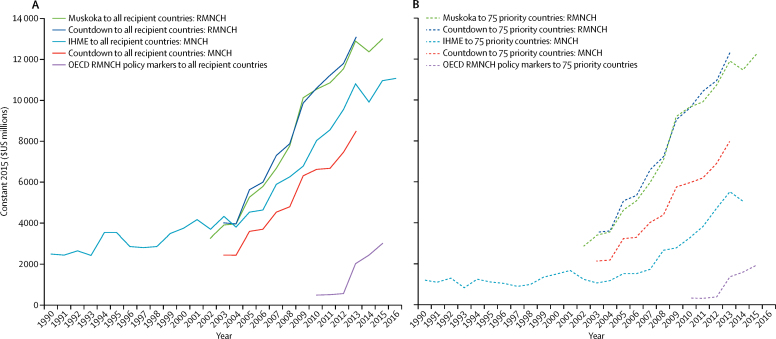
Estimates of aid for RMNCH, 1990–2016 Different methods indicate different levels but similar trends in global aid. (A) Findings for all recipient countries. (B) Findings for the 75 priority countries as a group. RMNCH=reproductive, maternal, newborn, and child health. MNCH=maternal, newborn, and child health. IHME=Institute for Health Metrics and Evaluation. OECD=Organisation for Economic Co-operation and Development.

All four approaches showed a large increase over time in aid for RMNCH, but their estimates of aid amounts differed by billions of dollars. Estimates for 2013 varied from $13·1 billion (Countdown RMNCH), $12·9 billion (Muskoka RMNCH), $10·8 billion (IHME MNCH), and $8·5 billion (Countdown MNCH), to just $2·0 billion (RMNCH policy marker; figure 1). RMNCH estimates from Muskoka and Countdown were highest and generally very similar. MNCH estimates from IHME were higher than those for Countdown in every year for which both produced estimates (2003–13) and were higher than both Muskoka and Countdown RMNCH estimates in 2002 and 2003. The OECD RMNCH policy marker generated the lowest estimates.

Estimates of aid for RMNCH for the 75 priority countries in 2013 varied from $12·2 billion (Countdown RMNCH) and $12·0 billion (Muskoka RMNCH), to $7·9 billion (Countdown MNCH), $5·5 billion (IHME MNCH), and $1·3 billion (RMNCH policy marker; figure 1). Unlike estimates for all recipients, the IHME MNCH estimates for the 75 priority countries were substantially lower than the Countdown MNCH estimates in all years for which both produced estimates (2003–13). For each year reported, Countdown indicated that the 75 priority countries received 88–93% of total aid for RMNCH and 88–92% of total aid for MNCH. Similarly, Muskoka indicated that the 75 priority countries received 87–94% of total aid for RMNCH. By contrast, the policy marker indicated that the 75 priority countries received 61–66% of all aid for RMNCH, and IHME indicated the 75 priority countries received just 24–51% of all aid for MNCH (appendix).

Annual rates of change differed substantially between approaches in some years. For example, from 2007 to 2008, Muskoka indicated a 16% increase in aid for RMNCH to all recipients, whereas the two Countdown estimates and IHME indicated 7–8% increases (appendix). For 2008–09, IHME indicated a 9% increase, compared with an increase of 25–31% with the other approaches. For 2013–14, the policy marker indicated a 23% increase, compared with a 4% increase with Muskoka and an 8% increase with IHME (appendix).

**Figure 2:**
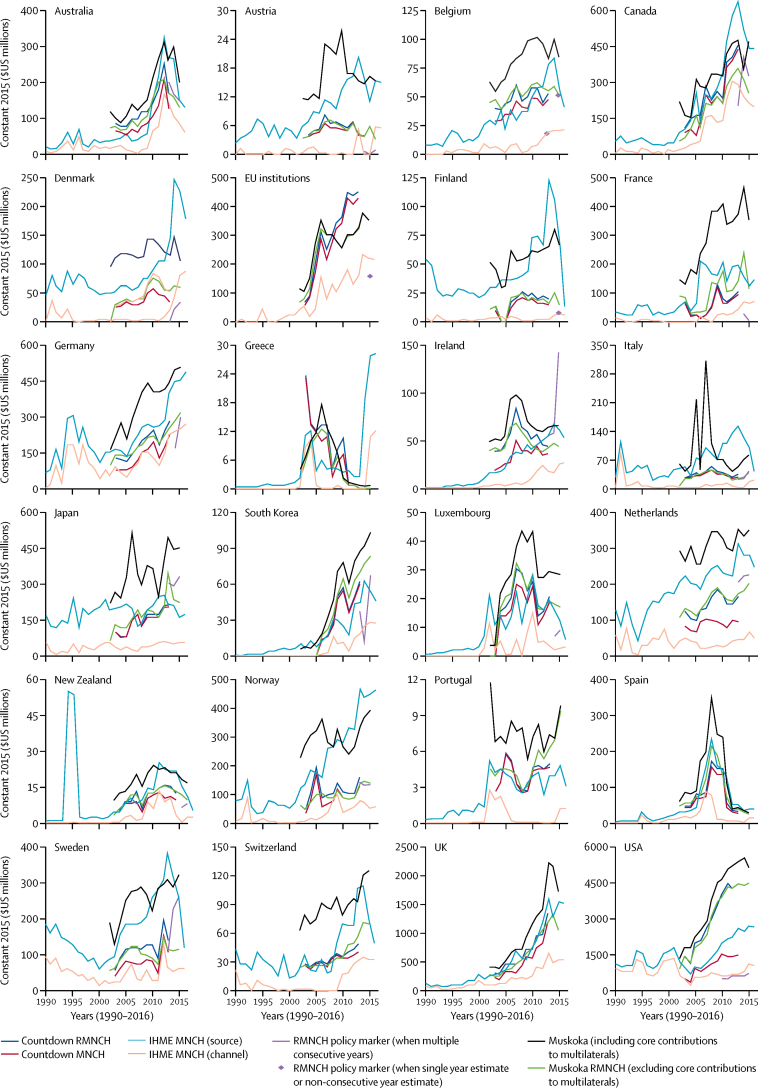
Estimates of aid for RMNCH for 1990–2016 for each of the 24 bilateral donors Different methods indicate different levels and trends in aid for RMNCH from individual donors. The 24 donors are longstanding members of the OECD Development Assistance Committee. EU=European Union. RMNCH=reproductive, maternal, newborn, and child health. MNCH=maternal, newborn, and child health. IHME=Institute for Health Metrics and Evaluation. OECD=Organisation for Economic Co-operation and Development.

Rankings, estimates, and trends for individual donors also differed substantially across approaches (figure 2; appendix). The USA was consistently the largest contributor of aid for RMNCH (appendix). However, estimates of US aid for RMNCH in 2013 varied from $5·4 billion (Muskoka RMNCH, including core multilateral contributions) and $4·5 billion (Muskoka RMNCH, excluding core contributions, and Countdown RMNCH), to $2·6 billion (IHME MNCH by source), $1·5 billion (Countdown MNCH), $0·7 billion (IHME MNCH by channel), and $0·6 billion (RMNCH policy marker). Only 12 of the 24 donors on which we focused applied the policy marker in all three agreed years (2013–15; Australia, Austria, Canada, Ireland, Italy, Japan, Korea, the Netherlands, Norway, Portugal, Sweden, and USA) and two did not apply it at all (Switzerland and UK).

**Figure 3:**
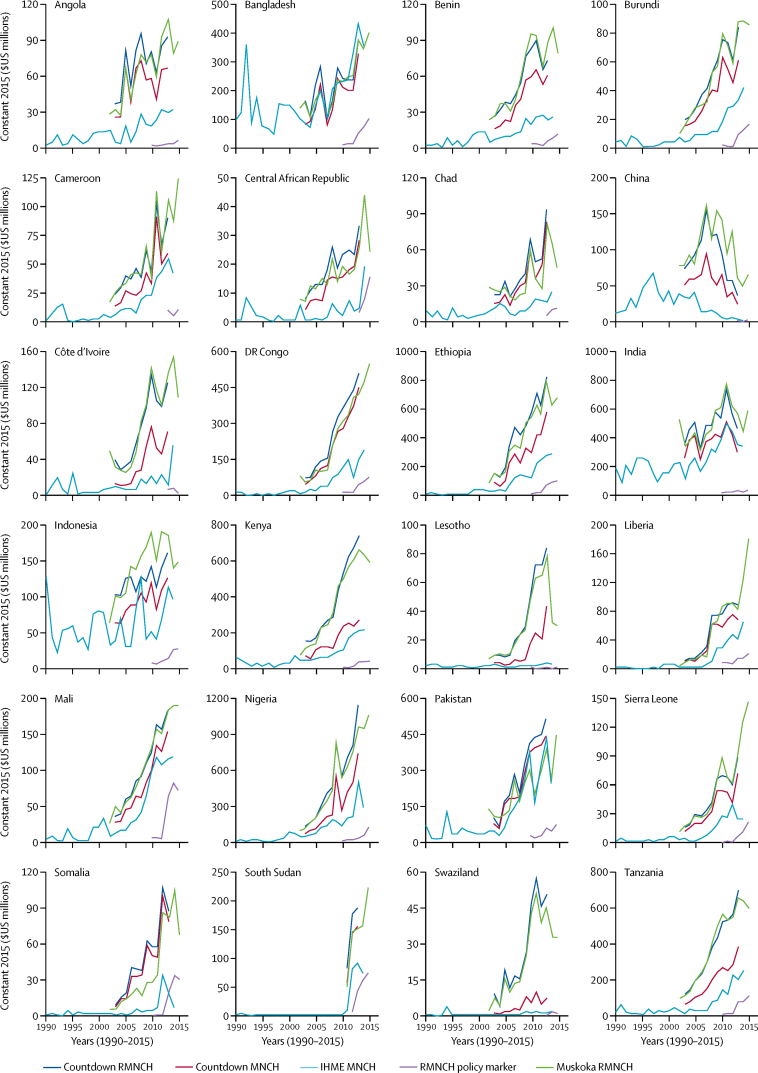
Estimates of aid for RMNCH for 1990–2015 for each of the 24 recipient countries Different methods indicate different levels and trends in aid for RMNCH for individual recipient countries. No recipient-level data were available for 2016 from any of the four approaches at the time of our analyses. The 24 recipient countries reflect the nine countries with the worst levels in 2013 in each of five indicators:^45^ maternal mortality ratio, number of maternal deaths, mortality rate in children younger than 5 years, number of deaths in children younger than 5 years, and female life expectancy. RMNCH=reproductive, maternal, newborn, and child health. MNCH=maternal, newborn, and child health. IHME=Institute for Health Metrics and Evaluation. OECD=Organisation for Economic Co-operation and Development.

For the 24 individual recipient countries on which we focused, the policy marker consistently resulted in the lowest aid estimates and IHME estimates tended to be the second lowest (figure 3). Muskoka and the two Countdown estimates were often similar, but the degree of similarity in aid levels and trends varied across recipients. For example, Muskoka and Countdown indicated substantial increases in funding over time for Angola, Chad, DR Congo, Lesotho, Somalia, and Swaziland, whereas IHME indicated consistently low funding for these countries (figure 3). We noted a similar positive relationship between RMNCH aid per child aged less than 5 years and child mortality for Muskoka and Countdown, but a much flatter relationship for IHME and the policy marker, involving extreme outliers (appendix).

To understand why the four approaches produced different estimates, we first examined their objectives. The Countdown, Muskoka, and policy marker approaches were developed to assess aid supporting RMNCH, whereas the IHME approach was developed to assess aid flows to the health sector and to characterise these flows by policy focus area (panel). This difference of objective affected how each approach distinguished aid for RMNCH from aid for other purposes, which in turn explained some of the differences observed in estimates and trends. However, there were also many further differences in methods—which we describe below—that were unrelated to this difference of objective and led to substantial differences in estimates.

All four approaches used the OECD Creditor Reporting System database as the main or only data source (table 1). Countdown supplemented the Creditor Reporting System with data obtained from Gavi, the vaccine alliance, on its disbursements in 2003–06. For estimates by donor, Muskoka used additional OECD data on donors' core contributions to multilateral institutions. IHME made more substantial use of additional data sources. To estimate aid from donor countries and the EU, IHME combined the OECD Creditor Reporting System database with additional OECD data for 1990–2014 and used donor budgets for 2015–16. For other donors, IHME used many other data sources (appendix).^46^

**Table 1:** Summary of key analytical choices

	**Countdown**	**Muskoka**	**OECD RMNCH policy marker**	**IHME**
What time period do the estimates analysed in this Article cover?	2003–13	2002–15 (presented in this Article); 2006–14 (PMNCH reports)	2013–15 (donor reporting requested); 2010–15 (US reporting)	1990–2016 (global and donor-specific); 1990–2014 (recipient-specific)
Which aid data sources are used?	OECD's Creditor Reporting System database; missing data from Gavi, the vaccine alliance for 2003–06, replaced with data obtained directly from Gavi	OECD's Creditor Reporting System database; for donor-specific estimates, Creditor Reporting System supplemented with additional OECD data tables on core contributions to multilaterals	OECD's Creditor Reporting System database	For 23 donor countries and the EU, OECD Creditor Reporting System and Development Assistance Committee databases were combined; for other donors, institutions' financial reports, audited financial statements, direct correspondence, and online databases; US tax filings; the Foundation Center's grants database; and the annual report on charities registered with the US Agency for International Development were used^46^
Which flow types are included?	Official development assistance and private grants	Official development assistance	Official development assistance (required to be coded) plus other official flows (optional to be coded)	Official development assistance, private grants, and donor administration costs
Which donors' aid is included?	Data from all 84 donors reporting to Creditor Reporting System evaluated; 51 donors (31 countries and 19 multilateral institutions and the Bill & Melinda Gates Foundation) considered to have provided aid for RMNCH based on Countdown criteria	Data from all 84 donors reporting to Creditor Reporting System evaluated; 63 donors (38 countries and 25 multilateral institutions) considered to have provided aid for RMNCH based on Muskoka criteria	All 84 donors reporting to Creditor Reporting System asked to code data on their official development assistance (if any) and other official flows (if any) for 2013 onwards; 33 donors (29 countries plus four multilateral institutions) coded any data with at least one non-zero value based on RMNCH policy marker criteria	Data from 36 of the 84 donors reporting to Creditor Reporting System evaluated using either Creditor Reporting System or other data sources; 34 of these 84 donors (24 countries plus nine multilateral institutions and the Gates Foundation) considered to have provided aid for RMNCH based on IHME criteria; additionally, IHME evaluated data from Pan American Health Organisation, >1000 foundations, and >500 non-governmental organisations based in the USA, and from >100 international non-governmental organisations registered in the USA; numbers of these considered to have provided aid for RMNCH based on IHME criteria is unclear
How are RMNCH activities defined? (see appendix for details)	Broadly	Broadly	Broadly	Narrowly
How is aid for RMNCH distinguished from other aid?	Analysts code each record individually according to one of 27 codes in an activity-based RMNCH framework; depending on the code assigned, a combination of assumptions or year-specific and recipient country-specific financing, and health and demographic data define the proportion of the record's value (0–100%) categorised as supporting RMNCH	Analysts categorise a proportion (0–100%) of the value of each record as supporting RMNCH based on the record's Creditor Reporting System purpose code; the proportion associated with each purpose code reflects a combination of assumptions and data on financing, health, and demography in 2009 averaged across 49 low-income countries	Donors code each record to indicate whether approximately 0%, 25%, 50%, 75%, or 100% of the funding supports RMNCH; analysts can use these codes to generate estimates	Analysts combine existing categories (eg, Creditor Reporting System purpose codes and multilateral institutions' internal classification systems) with key term searches of descriptive text fields to classify funding as focused on either MNCH or other health focus areas (HIV, tuberculosis, malaria, other infectious diseases, health systems, non-communicable diseases, and other); where key terms indicate more than one health focus area for a record, its value is divided across focus areas in proportion to the number of key terms identified for each focus area
Are donor countries credited for their relevant contributions to multilateral institutions' core budgets?	Some; donor countries credited for relevant contributions to multilaterals for which the recipient and purpose are specified, but not for core contributions to multilaterals' general budgets	Mostly, donor countries credited for relevant earmarked contributions and for relevant core contributions to ten multilaterals; core contributions to the EU and other multilaterals not credited	As for Countdown	Yes; all aid flows traced back to a government, corporate, or private source
How is aid to unspecified, global, and regional recipients treated?	Included in estimates for each recipient country and the 75 priority countries	As for Countdown	Excluded from estimates for each recipient country and the 75 priority countries	Regional funding included, and both unspecified and global funding excluded from estimates for each recipient country and the 75 priority countries
How are currency values adjusted for inflation and exchange rates?	Used OECD methods: first adjusted for inflation in each donor country, then converted to $US using average exchange rates in a single year (2015 for our analysis)	As for Countdown	As for Countdown and Muskoka	First converted each year's aid to $US using average annual exchange rates, then applied US gross domestic product deflators, which account for inflation in the USA
How are estimates adjusted for under-reporting and reporting lags?	Not adjusted	Not adjusted; reporting lags addressed in text of PMNCH reports by providing indication of more recent trends in aid based on interviews with key donors	Not adjusted	For earlier years, used commitments to estimate disbursements and inflated detailed Creditor Reporting System data to match aggregate Development Assistance Committee data; for the decade to 2014, minor adjustments to disbursements to match reported commitments; for the most recent 2 years, generated estimated disbursements using regression models

OECD=Organisation for Economic Co-operation and Development. RMNCH=reproductive, maternal, newborn, and child health. IHME=Institute for Health Metrics and Evaluation. PMNCH=Partnership for Maternal, Newborn & Child Health. MNCH=maternal, newborn, and child health. EU=European Union.

Official development assistance was the main or only aid flow tracked by each of the four approaches (table 1). The OECD defines official development assistance as funds that promote economic development and welfare in low-income and middle-income countries. Muskoka only tracked official development assistance. The policy marker also tracked other official flows, but these funds had a negligible effect on its RMNCH aid estimates. In addition to official development assistance, Countdown also tracked private grants (exclusively from the Gates Foundation), which comprised 5–6% of its RMNCH estimates for 2009–13, the years for which the Gates Foundation reported its grants to the Creditor Reporting System. IHME tracked official development assistance, private grants, and donor administration costs.^39^ Gates Foundation funding comprised 5% of IHME estimates and including donor administrative costs increased IHME aid estimates by 9% for 1990 and by 14% for 2015 (IHME did not provide data tables on estimates of in-kind contributions, although this has been included in IHME's latest report,^36^ so these proportions were estimated from a bar graph).

The four approaches assessed aid from different donors. Muskoka, Countdown, and the policy marker assessed aid from all 86 donors reporting to the Creditor Reporting System (table 1). IHME assessed funding from 36 of these donors, and additional donors whose aid was not captured in the Creditor Reporting System. The 50 donors excluded from the IHME assessment accounted for only 1–2% of Muskoka and Countdown RMNCH estimates, and the additional donors assessed by IHME accounted for 7% of the IHME MNCH estimate in 2013.

Some initiatives excluded funding from large donors, which drove substantial differences in estimates. For example, the Muskoka method excluded private grants such as Bill & Melinda Gates Foundation funding; IHME excluded all disbursements from UNAIDS and the Global Fund from its MNCH estimates; and the OECD policy marker approach omitted major donors that did not use the policy marker, including the UK, which alone accounted for around 10% of Muskoka and Countdown RMNCH estimates in 2013.

To compare trends in the real value of money over time, the nominal values disbursed need to be adjusted to account for inflation (or deflation). Data must also be converted to a common currency. Countdown, Muskoka, and the policy marker converted disbursements using the OECD Development Assistance Committee deflators, a set of year-specific and donor-specific parameters that account for inflation in each donor's economy and changes in exchange rates over time. IHME converted all currencies to $US for each year and then applied the USA gross domestic product deflator, which accounts for inflation in the US economy, to all funding. This process led to substantial differences in estimated aid levels and trends for all donors (except the USA). At the extremes, IHME estimates for Japan were up to 133% higher (in 1995) than they would have been had the Development Assistance Committee deflators been used, and estimates for Australia ranged from 38% lower (in 2001) to 44% higher (in 2011; appendix).

**Figure 4:**
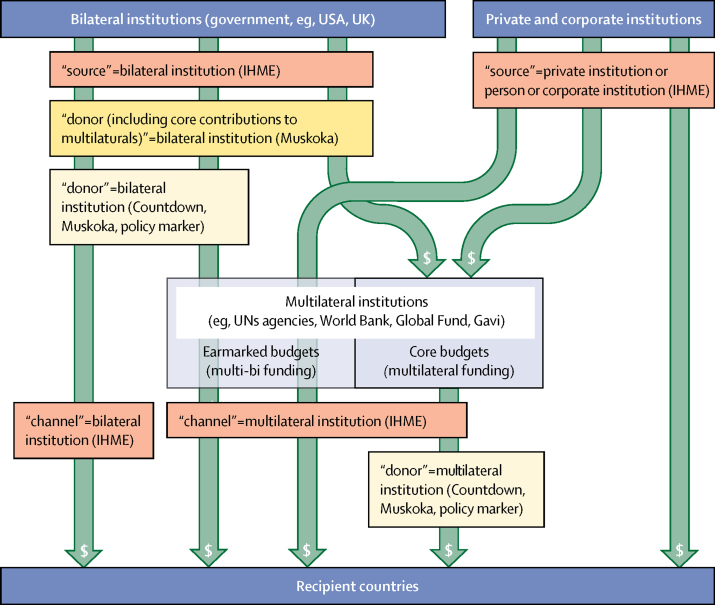
Different categorisations of multilateral and bilateral funding Funding flows from bilateral institutions and private and corporate institutions to recipient countries either directly or via multilateral institutions. The four approaches grouped and labelled these flows in different ways, which restricted the comparability of estimates of aid from individual countries or institutions across the different approaches. Adapted from the OECD^47^ and IHME.^23^ OECD=Organisation for Economic Co-operation and Development. IHME=Institute for Health Metrics and Evaluation.

Estimates of aid from individual donors (figure 2) were substantially affected by how each approach categorised the two types of funding that donor countries provide to multilateral institutions (eg, UN agencies, the World Bank, Gavi, and the EU; figure 4). Donor countries fund core budgets of multilaterals, which cover administration and activities directed by the multilateral, and provide earmarked funds, which allow the donor country to retain control over how funds are spent. IHME estimates of funding by source included both types of funding for multilaterals, whereas IHME estimates by channel included neither type of multilateral funding (including only direct bilateral expenditure). Countdown, Muskoka, and policy marker estimates by donor included earmarked but not core funding for multilaterals, meaning that they were not comparable with either of the IHME estimates. Muskoka's additional estimates by donor including core contributions to multilaterals^31^ and IHME's estimates by source both included the two types of support for multilateral institutions. The Muskoka approach indicated that core contributions to multilaterals constituted between 18% (USA) and 71% (Finland) of aid for RMNCH from the 23 donor countries on which we focused (figure 2; appendix). IHME indicated that core and earmarked contributions together constituted between 50% (Germany) and 94% (Finland) of aid for MNCH from the same countries over the same period (2002–15) (figure 2; appendix).

Estimates of the aid disbursed to individual recipient countries (figure 3) were substantially affected by how each approach treated aid disbursed to regions (eg, sub-Saharan Africa) and unspecified recipients. Countdown and Muskoka included a share of these regional and unspecified disbursements within estimates for individual recipient countries. Regional and unspecified disbursements thus constituted more than 20% of Countdown and Muskoka estimates for individual recipients and the 75 priority countries.

By contrast, IHME included regional disbursements but excluded disbursements to global and unspecified recipients from its estimates for individual recipient countries. The policy marker excluded disbursements to regional and unspecified recipients from their country-specific estimates. These exclusions reduced policy marker country-specific estimates by around 30% and IHME estimates by around 55% on average (figure 1; appendix).

Muskoka, Countdown, and the policy marker reported estimates based on donors' published disbursements, whereas IHME made substantial adjustments to account for donors' under-reporting and reporting lags. These adjustments allowed IHME to report aid estimates over a much longer period. They also affected IHME estimates for the years when the other approaches also reported estimates, although adjustments for the decade through 2014 were reported to be small. How IHME projections for 2015–16 compared with data reported subsequently is not clear.

The four approaches also differed in how they distinguished aid for RMNCH from aid for other purposes. We highlight differences in how each approach conceptualised aid for RMNCH and defined specific activities as relevant to RMNCH or not, and how this affected estimates.

Countdown, Muskoka, and the policy marker categorised aid as either supporting RMNCH or not. They defined RMNCH as a combination of demographic groups and health conditions and included activities not described as RMNCH, including investments in health conditions (such as HIV and other infectious diseases), health systems, and outside the health sector, if they were considered to support RMNCH directly. IHME divided the total value of aid for the health sector into mutually exclusive categories,^48^ and estimated the monetary value of health sector aid explicitly focused on MNCH, excluding aid targeting diseases, the health system, or other sectors, even if it directly benefited MNCH. For example, Muskoka, Countdown, and the policy marker would consider a project addressing malaria in pregnancy to support RMNCH, but IHME would require that the disbursement be assigned to maternal health or malaria or be divided between them; the full value could not be counted towards both maternal health and malaria estimates.

The policy marker had the broadest definition of RMNCH activities (appendix). Muskoka excluded research and the humanitarian sector, and Countdown excluded research and the water and sanitation sector. IHME had the narrowest definition, excluding HIV and other sexually transmitted infections, malaria, water and sanitation, funding from the USA's National Institutes of Health, and humanitarian aid from its MNCH estimates. IHME included family planning in its MNCH definition, whereas Countdown classified family planning within RMNCH but not MNCH.

The differing treatment of HIV funding across approaches explained much of the difference in estimates and trends for several high-burden recipient countries (notably Lesotho and Swaziland) and for the USA, and thus some of the differences in overall estimates. IHME and Muskoka's exclusion of humanitarian funding accounted for their consistently lower estimates for several conflict-affected countries (notably Central African Republic and Somalia) compared with Countdown's MNCH and RMNCH estimates (appendix).

**Figure 5:**
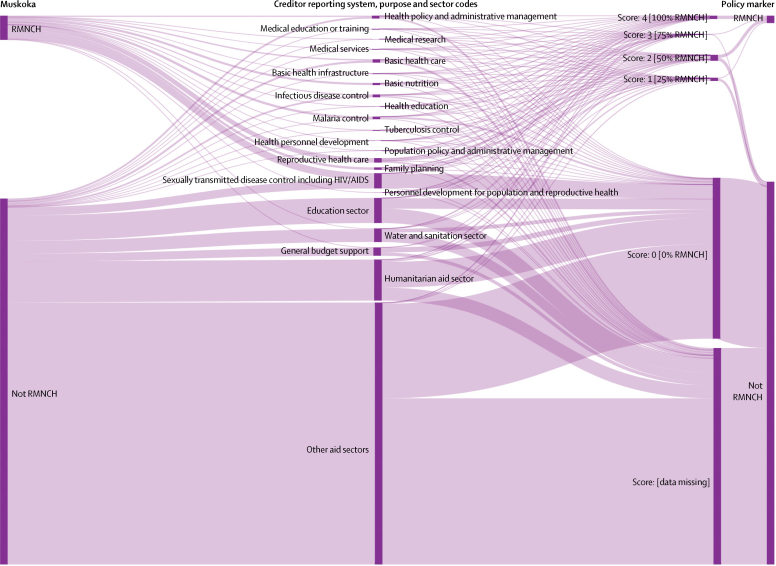
Creditor Reporting System purpose codes *vs* Muskoka and the OECD RMNCH policy marker to classify aid for RMNCH and other purposes, 2013–15 Sankey diagram showing how the same funding flows are categorised by the Creditor Reporting System sector and purpose codes, the Muskoka initiative, and the OECD RMNCH policy marker. Data in this diagram reflect all 2013–15 official development assistance flows from 24 bilateral donors (including the EU) in the June, 2017, version of the Creditor Reporting System database. OECD=Organisation for Economic Co-operation and Development. RMNCH=reproductive, maternal, newborn, and child health.

Each approach used a different set of techniques to distinguish aid for RMNCH from aid for other purposes. As all approaches used the OECD Creditor Reporting System as their main or only data source, they all built on the structure of this system. Each Creditor Reporting System record reflects a disbursement for which a donor (including countries, multilateral institutions, and the Gates Foundation) has provided the value, recipient, year, and a text description of the funded activities, as well as other data. Donors must assign to each record a single Creditor Reporting System purpose code, which identifies the sector (eg, health or education) and the more specific development objective of the disbursement (figure 5).^39^

For the policy marker, donors were expected to provide additional data on every record they reported to the Creditor Reporting System for data from 2013 onwards. Donors needed to assign each record an integer score (0–4), indicating that approximately 0%, 25%, 50%, 75%, or 100% of the value of the record supported RMNCH.^40^

IHME classified health sector aid into ten health focus categories and 34 subcategories using several automated algorithms. Aid flowing through multilaterals was classified either within a single category (eg, UNICEF as newborn and child health) or by using the institution's internal classification system or key term searches. Aid provided directly from donor countries and the EU to recipient countries was classified using key term searches and Creditor Reporting System purpose codes. In cases where a record contained key terms for more than one of IHME's categories, its value was divided across categories in proportion to the number of key terms for each category. A record described as prevention of vertical HIV transmission in the purpose code for sexually transmitted diseases including HIV/AIDS, for example, would be categorised in full to IHME's HIV category and no portion of it would be counted towards IHME's MNCH categories (figure 5).

**Figure 6:**
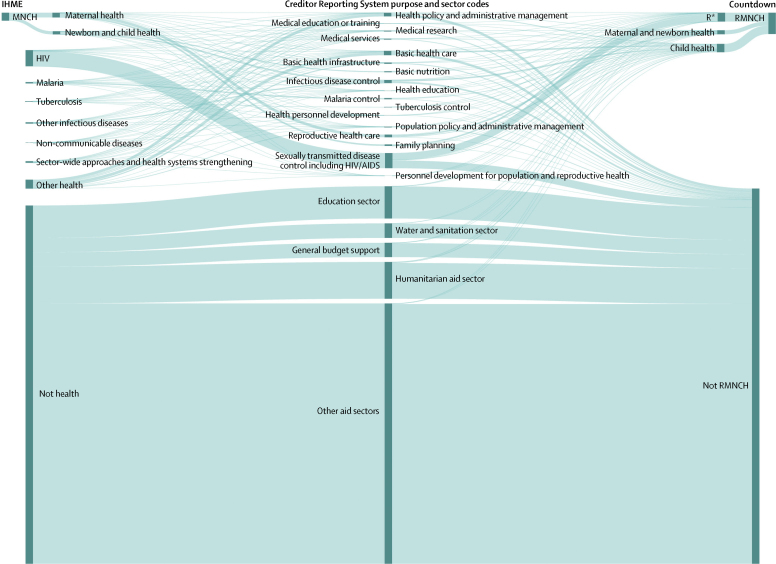
Creditor Reporting System purpose codes *vs* IHME and Countdown to classify aid for RMNCH and other purposes, 2003–13 Sankey diagram showing how the same funding flows are categorised by the Creditor Reporting System purpose and sector codes, the IHME, and the Countdown RMNCH aid tracking exercise. Data in this diagram reflect all 2003–13 official development assistance flows from 24 bilateral donors (including the EU) in the Countdown database. IHME procedures for allocating funding to different health sector categories were recreated based on their publications and personal communications. IHME=Institute for Health Metrics and Evaluation. RMNCH=reproductive, maternal, newborn, and child health. R*=family planning, sexual health, and sexually transmitted infections, including HIV.

Countdown analysts read the donor, purpose code, and descriptive text fields for each Creditor Reporting System record in all sectors and assigned one of 27 codes based on their relevance to RMNCH (figure 6). The proportion of the record's value counted towards RMNCH estimates reflected assumptions and year-specific and country-specific financing, health, and demographic data. For example, records coded as general funding for HIV were assumed to benefit child health in proportion to the share of children aged under 5 years in the population with HIV. The full value of projects to prevent malaria in pregnancy or vertical transmission of HIV were counted towards RMNCH (Figure 5, Figure 6).

The original Muskoka approach defined by the G8 counted a proportion of funding as supporting RMNCH based on the existing Creditor Reporting System purpose code. For example, 88·5% of each disbursement with the malaria control purpose code was considered to support RMNCH. These purpose code-based percentages reflected assumptions and financing, health, and demographic data for 2009 averaged across 49 low-income countries. Additionally, the Muskoka method also counted a proportion of core contributions from donor countries to ten multilateral institutions towards RMNCH estimates. For example, 55% of each donor's core contributions to UNICEF were counted toward estimates of its aid for RMNCH. These institution-based percentages reflected each multilateral institution's assessment of the proportion of its activities in 2009 that benefited RMNCH. Our donor-specific Muskoka estimates replicated this approach and extended it to the 24 donors on which we focused (figure 2). When the PMNCH generated Muskoka estimates of RMNCH disbursements for the 75 priority recipients and the world's 49 poorest countries, its analysts applied the purpose code-based fixed percentages to all donor disbursements in the Creditor Reporting System, which we replicated in generating global (figure 1) and recipient-specific estimates (figure 3).

When applying these procedures to identify aid for RMNCH within the same dataset, we found that the Muskoka approach classified 12% less overall funding as RMNCH than did Countdown (appendix). This proportion varied little over time (range 3–15), but varied across donors (–15 to 37) and the 24 recipient countries (–11 to 60; appendix). Countdown classified 44% (39 to 48) less overall funding to MNCH than to RMNCH. IHME classified 37% less funding as MNCH than did Countdown (and 65% less funding than Countdown had classified as RMNCH); this proportion varied substantially over time (range 24 to 56), and across donors (19 to 87%) and recipients (–31 to 95%; appendix).

## Discussion

The IHME, Muskoka, Countdown, and OECD policy marker approaches led to substantially different estimates of levels and trends in aid for RMNCH, especially for individual donors and recipient countries. These differences are large enough to lead to different conclusions about whether donors and recipient countries have fulfilled their obligations and commitments and about the adequacy, targeting, and effectiveness of aid. As conclusions regarding past aid efforts can have important ramifications for future global health policies and funding, it is important that policy makers, advocates, and academics understand and recognise the limitations of the estimates they use so that they can draw appropriate conclusions (table 2).

**Table 2:** Aims, appropriate uses, advantages, and disadvantages of the four approaches for estimating aid for RMNCH

	**Countdown**	**Muskoka**	**OECD RMNCH policy marker**	**IHME**
Aim	To estimate the monetary value of aid promoting RMNCH	As for Countdown	As for Countdown and Muskoka	To estimate the monetary value of development (not humanitarian) aid to the health sector and then to characterise the health focus areas of this aid
Appropriate uses of estimates of aid for (R)MNCH for each approach	Assess effectiveness of aid in improving coverage and health, assess adequacy of aid relative to cost estimates, granular donor-specific and recipient-specific analyses	Frequent global monitoring, more appropriate for global than donor-specific or recipient-specific analyses, assess adequacy of aid relative to cost estimates (especially at global level)	Limited to analyses of individual donors' aid flows because few donors have provided complete data	Analyse donors' priorities, eg, whether setting global goals led to changes in funding targeting RMNCH or specific diseases; granular donor-specific analyses of funding priorities
Advantages	Exploits publicly available data, provides relatively precise estimates based on available data	Quick to implement, exploits publicly available data, fully transparent, agreed by donors and generates estimates they can predict, credits donor countries for their contributions to most major multilateral institutions (but not the EU), adaptable to new goals, replicable	Fully transparent, agreed by donors and generates estimates they can predict, quick for analysts to implement, replicable estimates (although donor coding is not replicable)	Longest time trends; estimates development aid for health sector as a whole; adaptable to new goals; fully credits donor countries for their contributions to multilateral institutions, including the EU; exploits some descriptive data on individual projects (using key terms)
Disadvantages	Perceived subjectivity, complexity, labour-intensive to implement, open to human error in coding, not readily adaptable to new goals	Imprecise process for identifying aid; excludes humanitarian sector, so estimates of health aid biased against countries in crisis and donors focused on health in humanitarian contexts	Not readily adaptable to new goals; burdensome for donors; no robust trend analysis possible for global aid, recipients, or most donors, because of lack of data; relatively imprecise coding scheme; donors might code differently, making comparisons between donors problematic	Complexity; does not fully exploit publicly available data; excludes humanitarian sector, so estimates of health aid biased against countries in crisis and donors focused on humanitarian contexts

RMNCH=reproductive, maternal, newborn, and child health. OECD=Organisation for Economic Co-operation and Development. IHME=Institute for Health Metrics and Evaluation.

We identified important differences and limitations in how the four initiatives identified and analysed aid in general, which are relevant for any aid tracking initiative. All four used the OECD Creditor Reporting System database and none included bilateral aid from China, Russia, India, or Brazil. None of the four initiatives have yet exploited the AidData database, which has recently added data from China and other non-Development Assistance Committee donors.^49^ Converting funding flows to $US before accounting for inflation (and therefore assuming US inflation reflected inflation rates of all countries) substantially altered IHME estimates and trends and reflected neither the opportunity cost to donors (except the USA) of giving nor to recipients of receiving the given nominal flows in one year rather than another. IHME's complex methods of adjusting reported donor data for under-reporting and reporting lags allowed it to report estimates over a much longer period than the other approaches and might have improved the completeness of estimates, but the accuracy of these projections remains uncertain and such adjustments do not hold donors accountable for complete and accurate reporting. Estimates by recipient country were substantially affected by whether regional and unspecified allocations were included in estimates for individual countries. Estimates by donor were substantially affected by whether donor countries were credited for their support to multilateral institutions. Only Muskoka and IHME credited countries for their core funding to any multilateral institutions and only IHME credited European countries for aid provided through the EU; such crediting is important for ensuring that accountability mechanisms do not disincentivise support for multilateral cooperation and EU membership.

We also identified important differences and limitations in how each approach distinguished aid for RMNCH from aid for other purposes, which offer useful insights for tracking aid to any specific area. The four approaches had different objectives and so conceived of aid for RMNCH differently; Muskoka, Countdown, and the policy marker sought to estimate the monetary value of aid supporting RMNCH, whereas IHME sought to characterise the policy focus of aid to the health sector. As a result, IHME estimated the value of aid explicitly described as promoting RMNCH, excluding aid to diseases, the general health system, or non-health sectors, which substantially reduced its estimates relative to the other approaches. Donors often categorise health funding in emergency contexts as humanitarian rather than falling within the health sector, but IHME and Muskoka excluded humanitarian funding from their RMNCH estimates, which underestimated aid for RMNCH to crisis-affected countries. Despite increasing evidence of the effect on health investments in non-health sectors,^50^, ^51^ only Muskoka included a portion of funding to the water and sanitation sector, and none of the approaches included activities in education, transport, or social protection.

To monitor funding supporting each SDG and the Global Strategy for Women's, Children's, and Adolescents' Health, aid tracking methods should be built on a sound conceptual framework, which recognises that funding can simultaneously support multiple objectives.^16^ Consistent with the System of Health Accounts,^52^ such a framework must recognise that diseases and population groups necessarily overlap; funding oriented towards a category in one dimension (eg, health system functions) will also support categories in other dimensions (eg, diseases and population groups). For example, if a donor concentrated exclusively on addressing the crisis in human resources for health, its aid would simultaneously promote RMNCH and disease control efforts. This conceptualisation of aid supports adherence to aid effectiveness principles^53^ by decreasing pressure on donors to fund many separate projects, thereby reducing aid fragmentation and transaction costs, and increasing funding efficiency.^54^ This approach also promotes a multi-sectoral approach and recognises the interdependent, rather than competing, nature of the SDGs. This conceptualisation is also consistent with the Global Financing Facility's estimates of incremental financing needs for RMNCH and adolescent health (RMNCAH), which emphasise the role of malaria, HIV, and non-health sectors as key determinants of RMNCAH.^55^

Both IHME and Countdown used complex techniques to distinguish aid for RMNCH from other aid. Although their respective techniques were different (IHME's were automated, whereas Countdown researchers manually coded records), these technical differences had far less effect on estimates than differences in whether the approaches included funding for diseases and other objectives in their RMNCH estimates. The policy marker coding framework appeared both simple and reasonably precise; however, because it relied on donors for coding and half of major health donors did not apply it consistently, its estimates were low and uninformative. The Muskoka method used high-level assumptions without project-level coding or review, which meant that its global estimates, which were similar to Countdown's, were more reliable than its granular estimates (eg, by donor or recipient), which were more likely to be affected by misclassification. All four initiatives entailed many assumptions and none quantified the uncertainty in their estimates. To facilitate the classification of aid across multiple dimensions, some researchers have advocated for the OECD to adopt a matrix approach;^56^ however, the Creditor Reporting System purpose code framework remains ill adapted to tracking aid for specific population groups or health conditions.

Our comparative analysis has several limitations. First, our role in producing the Countdown estimates might raise questions of bias in our comparisons. Our intimate knowledge of the Countdown methods gives us unique insight into both its strengths and weaknesses, which we have endeavoured to elucidate fairly, and we do not intend to produce further estimates using this approach. Second, we focused on comparing overall RMNCH estimates and have not compared subcategories therein, either because subcategories were not available (Muskoka and policy marker), or because of differences in subcategory definitions (IHME and Countdown). Third, we sought to compare key methodological differences, but further analytical choices not explored here could also influence estimates.

We showed that tracking aid involves many analytical choices, which substantially affect estimates. Most of the choices made in the four approaches we compared were neither right nor wrong, but involved trade-offs between simplicity, timeliness, precision, accuracy, efficiency, flexibility, replicability, and the incentives created. We argue that the most appropriate methods depend on the objectives. The methods used for advocacy and accountability efforts should, we believe, prioritise timeliness (so findings are up to date), simplicity (so findings are transparent for donors and advocates), replicability (so findings are verifiable and donors can predict them), efficiency (so findings are not overly expensive to produce), flexibility (so tracking methods can be adapted to shifting advocacy objectives), and creating positive incentives for donors. Of the four methods we compared, Muskoka best reflected these priorities. Building on our analysis, the PMNCH and Countdown to 2030 convened stakeholders in 2018 and are engaged in ongoing consultation and analysis regarding how best to improve the precision and accuracy of the Muskoka approach, while maintaining its speed, replicability, and positive incentives for donors. The methods used for tracking aid for research purposes should, we argue, prioritise the accuracy and precision of estimates, with due regard for efficiency. Such in-depth analyses could include assessments of the effect of aid on health outcomes, examination of levels and trends in aid for narrow areas and at a granular level, or development and validation of quick and simple methods for advocacy and accountability exercises. For such research, combining key term searches and Creditor Reporting System purpose codes with manual review of a restricted set of records might provide a suitable balance between rigour and efficiency. All future approaches should explore and communicate uncertainty in estimates.
