# Supplementary material for: Tracking aid for global health goals: a systematic comparison of four approaches applied to reproductive, maternal, newborn, and child health
Source: Lancet Glob Health. 2018 Jul 14;6(8):e859–74. doi: 10.1016/S2214-109X(18)30276-6 (PMC6057137; doi:10.1016/S2214-109X(18)30276-6)
Supplement: Supplementary appendix [file mmc1.pdf]

THE LANCET
Global Health

Supplementary appendix

This appendix formed part of the original submission and has been peer reviewed. We post it as supplied by the authors.

Supplement to: Pitt C, Grollman C, Martinez-Alvarez M, Arregoces L, Borghi J. Tracking aid for global health goals: a systematic comparison of four approaches applied to reproductive, maternal, newborn, and child health. *Lancet Glob Health* 2018; **6:** e859–74.

Tracking aid for global health goals:

A systematic comparison of four approaches applied to reproductive, maternal, newborn, and child health

WEB APPENDIX: Supplementary findings

Catherine Pitt, Christopher Grollman, Melisa Martinez-Alvarez, Leonardo Arregoces, Josephine Borghi

CONTENTS

1. Supplementary findings: Estimates
    1.1. Comparison of levels and trends in aid for RMNCH across metrics: All recipients and 75 priority recipients
    1.2. Comparison of donor rankings in 2013 across metrics
    1.3. Relationships between aid for RMNCH per child and child mortality
2. Supplementary findings: Why were the estimates different?
    2.1. Aid data sources
    2.2. Donors
    2.3. Classifying bilateral and multilateral funding
    2.4. Adjustments for inflation and exchange rates
    2.5. Recipients
    2.6. Adjustments for underreporting and reporting lags
    2.7. How aid for RMNCH was distinguished from aid for other purposes
References

TABLES

Supplementary Table S1 Levels and trends in aid for RMNCH: All recipients and 75 priority recipients (constant 2015 USD)

Supplementary Table S2 Bilateral donors reporting to the CRS: Inclusion by each approach for estimating aid for RMNCH

Supplementary Table S3 Multilateral and private donors reporting to the CRS: Inclusion by each approach for estimating aid for RMNCH

Supplementary Table S4 Muskoka methodology: Effects of including bilateral donors' core contributions to multilaterals in estimates of aid for RMNCH, 2002-15

Supplementary Table S5 Comparison of techniques within the Muskoka method for identifying multilateral aid for RMNCH

Supplementary Table S6 Magnitude of differences in IHME and OECD deflators

Supplementary Table S7 Effect of choice of deflators on IHME estimates

Supplementary Table S8 The 75 Countdown to 2015 priority recipients

Supplementary Table S9 Classification of specific services and activities in Creditor Reporting System (CRS) purpose codes and four RMNCH tracking initiatives

Supplementary Table S10 Comparison of the composition of estimates of aid for RMNCH in 2013 by sector and purpose code

Supplementary Table S11 Comparison of the composition of estimates of aid for RMNCH by sector and purpose code (replication in Countdown dataset for 24 bilateral donors)

FIGURES

Supplementary Figure S1 Comparison of donor rankings in 2013 by metric (constant 2015 USD)

Supplementary Figure S2 Comparison of donor rankings by metric, $0 to $650m (constant 2015 USD) in 2013

Supplementary Figure S3 Comparison of donor rankings by metric, $0 to $100m (constant 2015 USD) in 2013

Supplementary Figure S4 Relationships between aid for RMNCH per child and child mortality across the 75 priority recipient countries in 2013

Supplementary Figure S5 Disbursements to specified countries, regions, global, and unspecified recipients

Supplementary Figure S6 Muskoka and Countdown RMNCH estimates over time by purpose and sector codes

Supplementary Figure S7 Muskoka RMNCH estimates by CRS purpose and sector codes and by recipient country, 2002-15

Supplementary Figure S8 Countdown RMNCH estimates by CRS purpose and sector codes and by recipient, 2003-13

Supplementary Figure S9 Countdown vs IHME vs Muskoka to classify aid for RMNCH and other purposes, 2003–13

Supplementary Figure S10 Effects of differing RMNCH definitions and allocation procedures on estimates and trends in aid for RMNCH from 24 bilateral donors, 2003-2013

Supplementary Figure S11 Effects of differing RMNCH definitions and allocation procedures on estimates and trends in aid for RMNCH from 24 bilateral donors, 2003-2013

Supplementary Figure S12 Effects of differing RMNCH definitions and allocation procedures on estimates and trends in aid for RMNCH from 24 bilateral donors by recipient country, 2003-2013

Supplementary Figure S13 Effects of different RMNCH definitions and allocation procedures on the distribution of aid for RMNCH per child from 24 bilateral donors in 2013

1. Supplementary findings: Estimates

1.1. Comparison of levels and trends in aid for RMNCH across metrics: All recipients and 75 priority recipients

We present the four approaches' estimates of aid levels and trends over time for all recipient countries and for the 75 priority recipient countries as a group. This data was used to create **Figure 1** in the main text.

Supplementary Table S1 Levels and trends in aid for RMNCH: All recipients and 75 priority recipients (constant 2015 USD)

Year	1990	1991	1992	1993	1994	1995	1996	1997	1998	1999	2000	2001	2002	2003	2004	2005	2006	2007	2008	2009	2010	2011	2012	2013	2014	2015	2016
ESTIMATES																											
All recipients (i.e. "global" estimates)																											
Muskoka RMNCH	NA	NA	NA	NA	NA	NA	NA	NA	NA	NA	NA	NA	3,259	3,922	3,973	5,257	5,769	6,680	7,768	10,141	10,553	10,829	11,534	12,872	12,379	13,004	NA
Countdown RMNCH	NA	NA	NA	NA	NA	NA	NA	NA	NA	NA	NA	NA	NA	4,000	3,963	5,621	5,992	7,311	7,880	9,869	10,585	11,235	11,772	13,088	NA	NA	NA
Countdown MNCH	NA	NA	NA	NA	NA	NA	NA	NA	NA	NA	NA	NA	NA	2,455	2,445	3,620	3,725	4,524	4,823	6,290	6,633	6,686	7,443	8,493	NA	NA	NA
IHME MNCH	2,502	2,444	2,675	2,434	3,538	3,540	2,852	2,819	2,851	3,482	3,741	4,161	3,696	4,322	3,809	4,539	4,627	5,873	6,261	6,796	8,016	8,538	9,520	10,822	9,919	10,973	11,064
RMNCH policy marker	NA	NA	NA	NA	NA	NA	NA	NA	NA	NA	NA	NA	NA	NA	NA	NA	NA	NA	NA	NA	497	521	589	2,006	2,469	3,027	NA
75 priority recipients																											
Muskoka RMNCH	NA	NA	NA	NA	NA	NA	NA	NA	NA	NA	NA	NA	2,856	3,395	3,531	4,606	5,060	5,939	6,929	9,205	9,582	9,880	10,620	11,963	11,404	12,187	NA
Countdown RMNCH	NA	NA	NA	NA	NA	NA	NA	NA	NA	NA	NA	NA	NA	3,530	3,582	5,046	5,328	6,560	7,136	9,017	9,574	10,390	10,877	12,229	NA	NA	NA
Countdown MNCH	NA	NA	NA	NA	NA	NA	NA	NA	NA	NA	NA	NA	NA	2,135	2,204	3,249	3,288	4,009	4,345	5,724	5,935	6,155	6,846	7,913	NA	NA	NA
IHME MNCH	1,223	1,093	1,289	823	1,270	1,099	1,026	892	972	1,333	1,510	1,690	1,232	1,051	1,220	1,495	1,513	1,706	2,679	2,740	3,287	3,752	4,656	5,501	5,053	NA	NA
RMNCH policy marker	NA	NA	NA	NA	NA	NA	NA	NA	NA	NA	NA	NA	NA	NA	NA	NA	NA	NA	NA	NA	317	327	360	1,330	1,582	1,911	NA
RATIO: Estimates for 75 priority recipients to global estimates																											
Muskoka RMNCH	NA	NA	NA	NA	NA	NA	NA	NA	NA	NA	NA	NA	88%	87%	89%	88%	88%	89%	89%	91%	91%	91%	92%	93%	92%	94%	NA
Countdown RMNCH	NA	NA	NA	NA	NA	NA	NA	NA	NA	NA	NA	NA	NA	88%	90%	90%	89%	90%	91%	91%	90%	92%	92%	93%	NA	NA	NA
Countdown MNCH	NA	NA	NA	NA	NA	NA	NA	NA	NA	NA	NA	NA	NA	87%	90%	90%	88%	89%	90%	91%	89%	92%	92%	93%	NA	NA	NA
IHME MNCH	49%	45%	48%	34%	36%	31%	36%	32%	34%	38%	40%	41%	33%	24%	32%	33%	33%	29%	43%	40%	41%	44%	49%	51%	51%	NA	NA
RMNCH policy marker	NA	NA	NA	NA	NA	NA	NA	NA	NA	NA	NA	NA	NA	NA	NA	NA	NA	NA	NA	NA	64%	63%	61%	66%	64%	63%	NA
YEAR-ON-YEAR % CHANGE																											
All recipients (i.e. "global" estimates)																											
Muskoka RMNCH	NA	NA	NA	NA	NA	NA	NA	NA	NA	NA	NA	NA	NA	20%	1%	32%	10%	16%	16%	31%	4%	3%	7%	12%	-4%	5%	NA
Countdown RMNCH	NA	NA	NA	NA	NA	NA	NA	NA	NA	NA	NA	NA	NA	NA	-1%	42%	7%	22%	8%	25%	7%	6%	5%	11%	NA	NA	NA
Countdown MNCH	NA	NA	NA	NA	NA	NA	NA	NA	NA	NA	NA	NA	NA	NA	0%	48%	3%	21%	7%	30%	5%	1%	11%	14%	NA	NA	NA
IHME MNCH	NA	-2%	9%	-9%	45%	0%	-19%	-1%	1%	22%	7%	11%	-11%	17%	-12%	19%	2%	27%	7%	9%	18%	7%	12%	14%	-8%	11%	1%
RMNCH policy marker	NA	NA	NA	NA	NA	NA	NA	NA	NA	NA	NA	NA	NA	NA	NA	NA	NA	NA	NA	NA	NA	5%	13%	241%	23%	23%	NA
75 priority recipients																											
Muskoka RMNCH	NA	NA	NA	NA	NA	NA	NA	NA	NA	NA	NA	NA	NA	19%	4%	30%	10%	17%	17%	33%	4%	3%	7%	13%	-5%	7%	NA
Countdown RMNCH	NA	NA	NA	NA	NA	NA	NA	NA	NA	NA	NA	NA	NA	NA	1%	41%	6%	23%	9%	26%	6%	9%	5%	12%	NA	NA	NA
Countdown MNCH	NA	NA	NA	NA	NA	NA	NA	NA	NA	NA	NA	NA	NA	NA	3%	47%	1%	22%	8%	32%	4%	4%	11%	16%	NA	NA	NA
IHME MNCH	NA	-11%	18%	-36%	54%	-13%	-7%	-13%	9%	37%	13%	12%	-27%	-15%	16%	22%	1%	13%	57%	2%	20%	14%	24%	18%	-8%	NA	NA
RMNCH policy marker	NA	NA	NA	NA	NA	NA	NA	NA	NA	NA	NA	NA	NA	NA	NA	NA	NA	NA	NA	NA	NA	3%	10%	270%	19%	21%	NA

1.2. Comparison of donor rankings in 2013 across metrics

We used slopegraphs[1] to illustrate the differences in donor rankings and estimates of each of 24 bilateral donors' aid for RMNCH across seven metrics. Given the highly skewed nature of the data, we present three separate graphs: A) $0 to $5,500m; B) $0 to $650m, and C) $0 to $100m, with each presenting the same data, increasingly zoomed in on lower-ranking donors. The United States was the leading donor by all metrics and was followed by the United Kingdom and Canada according to most, but not all metrics. Rankings for smaller donors varied more widely.

Supplementary Figure S1 Comparison of donor rankings in 2013 by metric (constant 2015 USD)

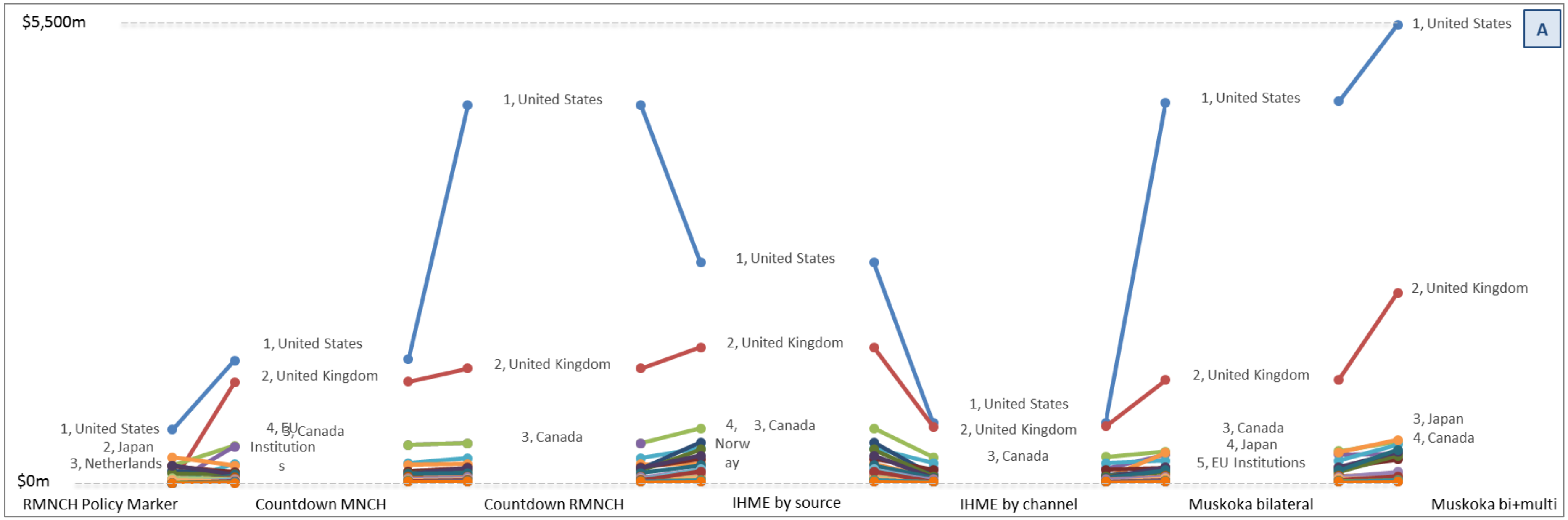

Supplementary Figure S2 Comparison of donor rankings by metric, $0 to $650m (constant 2015 USD) in 2013

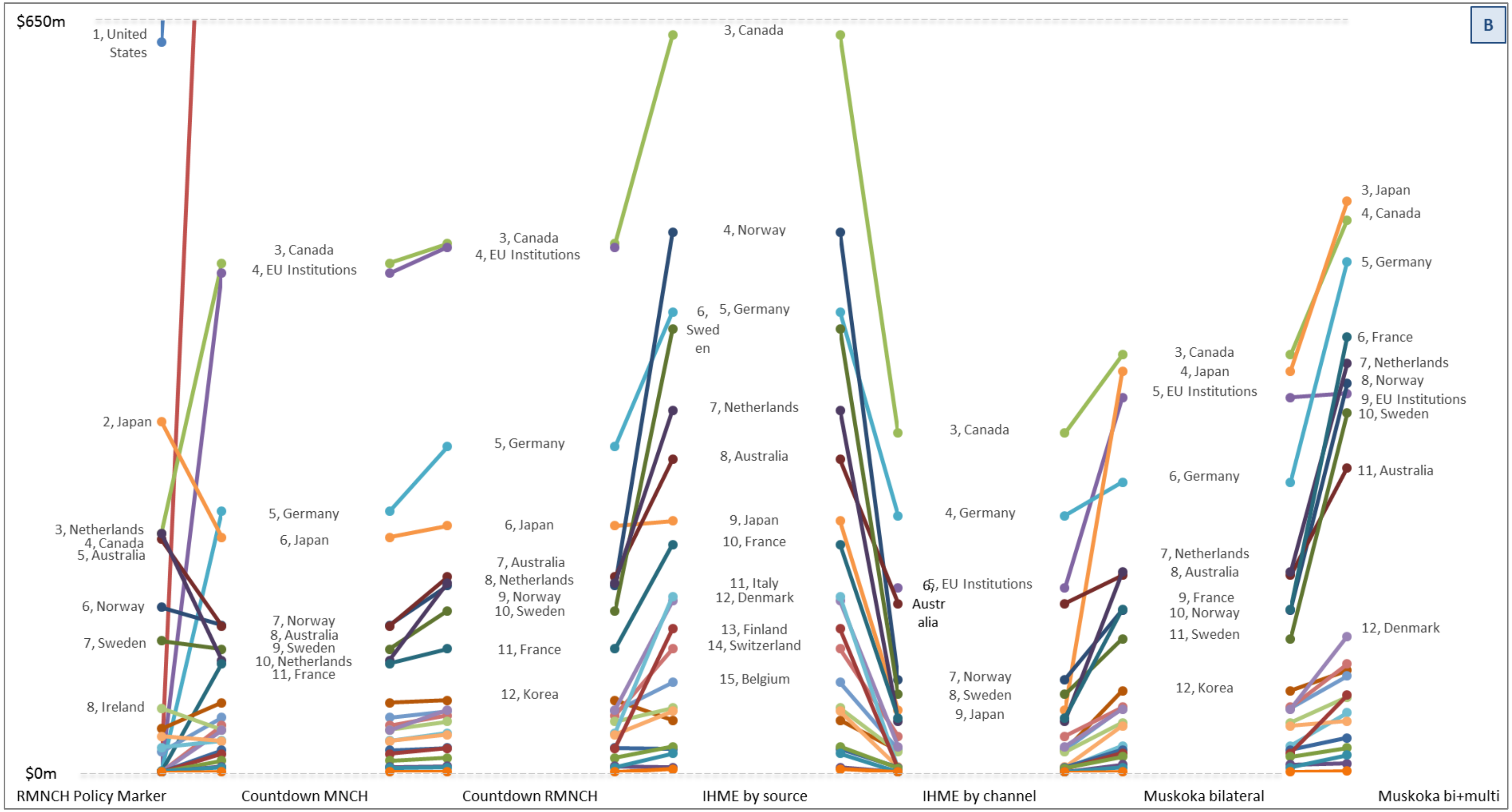

Supplementary Figure S3 Comparison of donor rankings by metric, $0 to $100m (constant 2015 USD) in 2013

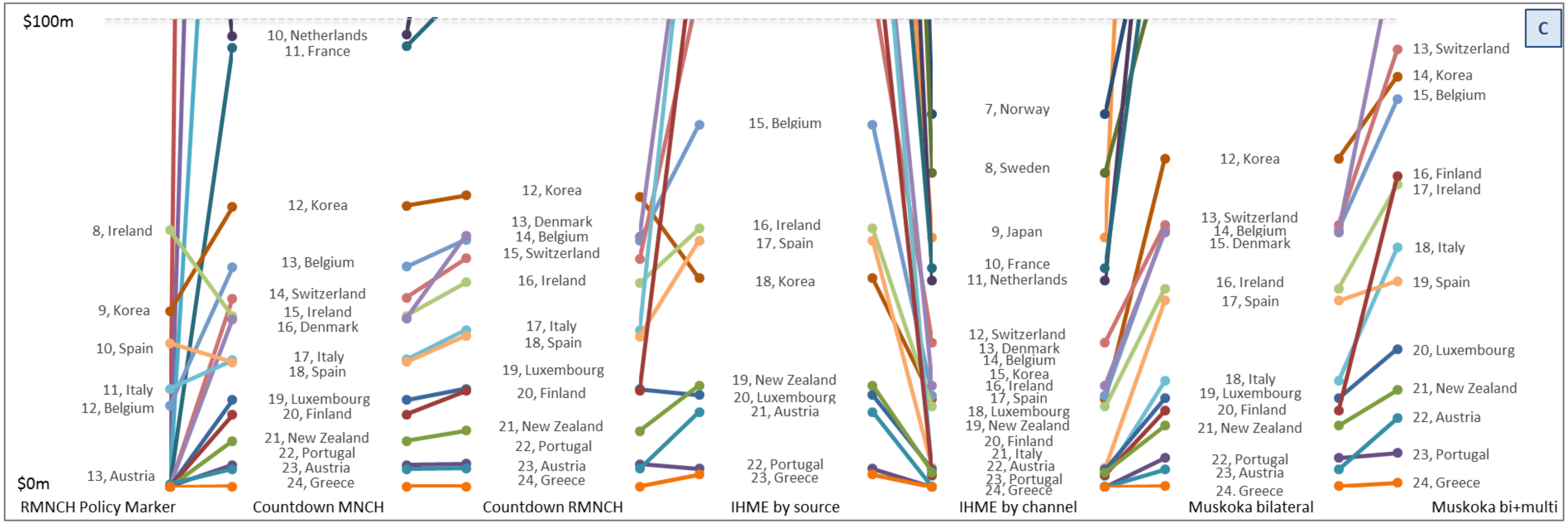

1.3. Relationships between aid for RMNCH per child and child mortality

We compared aid for RMNCH per child under 5 years [2] to the mortality rate of children under 5[3] using data for 2013 for each of the 75 priority countries. Estimates of aid for RMNCH reflect the most recent publicly available datasets for IHME[4] and Countdown[5] at the time of analysis and we produced new estimates based on the June, 2017, update of the OECD creditor reporting system database for the Muskoka method[6] and for the OECD policy marker.[7] RMNCH=reproductive, maternal, newborn, and child health. IHME=Institute for Health Metrics and Evaluation. OECD=Organization for Economic Co-operation and Development.

Supplementary Figure S4 Relationships between aid for RMNCH per child and child mortality across the 75 priority recipient countries in 2013

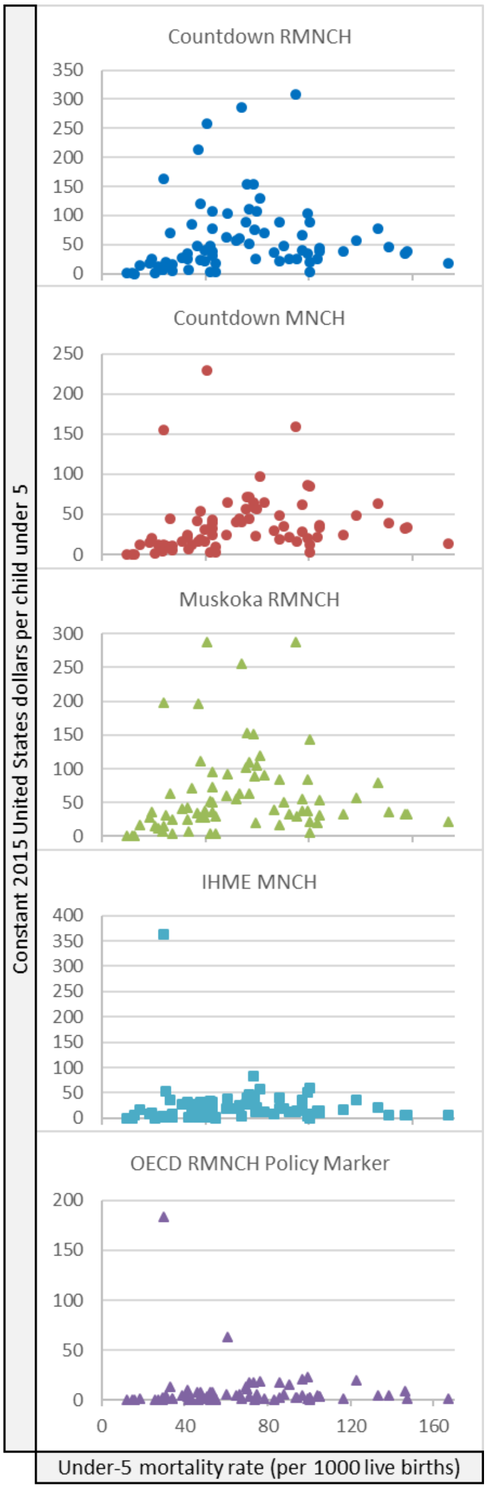

2. Supplementary findings: Why were the estimates different?

2.1. Aid data sources

The RMNCH policy marker is a variable within the OECD's CRS database, which was the primary data source for Countdown and Muskoka and the largest data source for IHME. Countdown supplemented CRS data with data obtained from GAVI for 2003-6, which was missing from the CRS. Global and recipient-specific Muskoka estimates were based solely on the CRS, however, generating donor-specific estimates required additional data on bilateral donors' core contributions to multilaterals, which we obtained from two OECD data tables: the DAC2a table and the "Imputed multilateral contributions to the health sector" table.

For 23 bilaterals and the EU, IHME combined the OECD's CRS data with data in the OECD-DAC aggregates database. For other donors, IHME obtained data directly from the institutions' own financial reports, audited financial statements, and online databases; direct correspondence with institutions; United States tax filings; the Foundation Center's grants database; and the annual report on charities registered with the United States Agency for International Development.[8]

2.2. Donors

Muskoka, Countdown, and the policy marker included data from all donors in the CRS. As of June 2017, the CRS contained data from 86 donors comprised of 30 bilateral DAC members, 20 bilateral non-DAC members, 35 multilaterals, and 1 private donor (the Gates Foundation). Muskoka and Countdown methods identified RMNCH disbursements from 65 and 51 donors, respectively. The RMNCH policy marker was coded with at least one non-zero value by 33 of these donors. IHME assessed funding from 36 of these donors and identified MNCH flows from 34 of them (24 bilaterals, 9 multilaterals, and Gates). Donors excluded from IHME's assessment accounted for 2% of Muskoka's estimate and 2% and 1% of Countdown's MNCH and RMNCH estimates, respectively (Supplementary Table S2, Supplementary Table S3).

In addition, IHME examined data from more than 1000 foundations (other than the Gates Foundation) and 500 NGOs based in the USA and from more than 100 international NGOs registered in the USA. Funds from these US foundations constituted 1% of IHME's MNCH estimate for 2013. Funds flowing through national and international NGOs and originating from corporate and other private sources (rather than bilaterals whose funding is tracked in the CRS) constituted 4% and 1%, respectively, of IHME's MNCH estimate. In addition, IHME also collected data from the Pan-American Health Organization (PAHO), which does not report to the CRS; funds channelled through PAHO constituted 0.3% of IHME's MNCH estimate in 2013. Together, these three groups and PAHO constituted 7% of IHME's MNCH estimate in 2013.

Supplementary Table S2 Bilateral donors reporting to the CRS: Inclusion by each approach for estimating aid for RMNCH

Donor and type	Any funding assessed for this donor?			Any OECD policy marker data coded					
	Countdown	Muskoka	IHME	2010	2011	2012	2013	2014	2015
BILATERAL DONORS									
Australia	Yes	Yes	Yes	No	No	No	Yes	Yes	Yes
Austria	Yes	Yes	Yes	No	No	No	Yes	Yes	Yes
Azerbaijan	Yes	Yes	No	No	No	No	No	No	No
Belgium	Yes	Yes	Yes	No	No	No	Yes	No	Yes
Bulgaria	Yes	Yes	No	No	No	No	No	No	No
Canada	Yes	Yes	Yes	No	No	No	Yes	Yes	Yes
Chinese Taipei	Yes	Yes	No	No	No	No	No	No	No
Croatia	Yes	Yes	No	No	No	No	No	No	No
Cyprus	Yes	Yes	No	No	No	No	No	No	No
Czech Republic	Yes	Yes	No	No	No	No	Yes	Yes	Yes
Denmark	Yes	Yes	Yes	No	No	No	Yes	Yes	Yes
Estonia	Yes	Yes	No	No	No	No	Yes	Yes	Yes
Finland	Yes	Yes	Yes	No	No	No	No	No	Yes
France	Yes	Yes	Yes	No	No	No	No	Yes	Yes
Germany	Yes	Yes	Yes	No	No	No	No	Yes	Yes
Greece	Yes	Yes	Yes	No	No	No	No	No	Yes
Hungary	Yes	Yes	No	No	No	No	No	No	No
Iceland	Yes	Yes	No	No	No	No	Yes	Yes	Yes
Ireland	Yes	Yes	Yes	No	No	No	Yes	Yes	Yes
Israel	Yes	Yes	No	No	No	No	No	No	No
Italy	Yes	Yes	Yes	No	No	No	Yes	Yes	Yes
Japan	Yes	Yes	Yes	No	No	No	Yes	Yes	Yes
Kazakhstan	Yes	Yes	No	No	No	No	No	No	No
Korea	Yes	Yes	Yes	No	No	No	Yes	Yes	Yes
Kuwait (KFAED)	Yes	Yes	No	No	No	No	No	No	No
Latvia	Yes	Yes	No	No	No	No	No	No	No
Liechtenstein	Yes	Yes	No	No	No	No	No	No	No
Lithuania	Yes	Yes	No	No	No	No	No	Yes	Yes
Luxembourg	Yes	Yes	Yes	No	No	No	No	Yes	Yes
Malta	Yes	Yes	No	No	No	No	No	No	No
Netherlands	Yes	Yes	Yes	No	No	No	Yes	Yes	Yes
New Zealand	Yes	Yes	Yes	No	No	No	No	Yes	Yes
Norway	Yes	Yes	Yes	No	No	No	Yes	Yes	Yes
Poland	Yes	Yes	No	No	No	No	Yes	Yes	Yes
Portugal	Yes	Yes	Yes	No	No	No	Yes	Yes	Yes
Romania	Yes	Yes	No	No	No	No	No	Yes	No
Russia	Yes	Yes	No	No	No	No	No	No	No
Saudi Arabia	Yes	Yes	No	No	No	No	No	No	No
Slovak Republic	Yes	Yes	No	No	No	No	Yes	No	Yes
Slovenia	Yes	Yes	No	No	No	No	No	No	Yes
Spain	Yes	Yes	Yes	No	No	No	Yes	Yes	No
Sweden	Yes	Yes	Yes	No	No	No	Yes	Yes	Yes
Switzerland	Yes	Yes	Yes	No	No	No	No	No	No
Thailand	Yes	Yes	No	No	No	No	No	No	No
Timor-Leste	Yes	Yes	No	No	No	No	No	No	No
Turkey	Yes	Yes	No	No	No	No	No	No	No
United Arab Emirates	Yes	Yes	No	No	No	No	No	No	No
United Kingdom	Yes	Yes	Yes	No	No	No	No	No	No
United States	Yes	Yes	Yes	Yes	Yes	Yes	Yes	Yes	Yes

Supplementary Table S3 Multilateral and private donors reporting to the CRS: Inclusion by each approach for estimating aid for RMNCH

Donor and type	Any funding assessed for this donor?			Any OECD policy marker data coded					
	Countdown	Muskoka	IHME	2010	2011	2012	2013	2014	2015
MULTILATERAL DONORS									
Adaptation Fund	Yes	Yes	No	No	No	No	No	No	No
African Development Bank	Yes	Yes	Yes	No	No	No	No	No	No
African Development Fund	Yes	Yes	Yes	No	No	No	No	No	No
Arab Bank for Economic Development in Africa	Yes	Yes	No	No	No	No	No	No	No
Arab Fund (AFESD)	Yes	Yes	No	No	No	No	No	No	No
AsDB Special Funds	Yes	Yes	Yes	No	No	No	No	No	Yes
Caribbean Development Bank	Yes	Yes	No	No	No	No	No	No	No
Climate Investment Funds	Yes	Yes	No	No	No	No	No	No	No
Council of Europe Development Bank	Yes	Yes	No	No	No	No	No	No	No
EU Institutions	Yes	Yes	Yes	No	No	No	No	No	Yes
Food and Agriculture Organisation	Yes	Yes	No	No	No	No	No	No	No
Global Alliance for Vaccines and Immunization	Yes	Yes	Yes	No	No	No	No	No	No
Global Environment Facility	Yes	Yes	No	No	No	No	No	No	No
Global Fund	Yes	Yes	Yes	No	No	No	No	No	No
Global Green Growth Institute	Yes	Yes	No	No	No	No	No	No	No
IDB Special Fund	Yes	Yes	Yes	No	No	No	No	No	No
IFAD	Yes	Yes	No	No	No	No	No	No	No
IMF (Concessional Trust Funds)	Yes	Yes	No	No	No	No	No	No	No
Inter-American Development Bank	Yes	Yes	Yes	No	No	No	No	No	No
International Atomic Energy Agency	Yes	Yes	No	No	No	No	No	No	No
International Development Association	Yes	Yes	Yes	No	No	No	No	No	No
International Labour Organisation	Yes	Yes	No	No	No	No	No	No	No
Islamic Development Bank	Yes	Yes	No	No	No	No	No	No	No
Montreal Protocol	Yes	Yes	No	No	No	No	No	No	No
Nordic Development Fund	Yes	Yes	No	No	No	No	No	No	No
OPEC Fund for International Development	Yes	Yes	No	No	No	No	No	No	No
OSCE	Yes	Yes	No	No	No	No	No	No	No
UN Peacebuilding Fund	Yes	Yes	No	No	No	No	No	No	No
UNAIDS	Yes	Yes	Yes	No	No	No	No	No	No
UNDP	Yes	Yes	No	No	No	No	No	No	No
UNECE	Yes	Yes	No	No	No	No	No	No	No
UNEP	Yes	Yes	No	No	No	No	No	No	No
UNFPA	Yes	Yes	Yes	No	No	No	No	No	No
UNHCR	Yes	Yes	No	No	No	No	No	No	No
UNICEF	Yes	Yes	Yes	No	No	No	No	No	No
UNRWA	Yes	Yes	No	No	No	No	No	No	No
WFP	Yes	Yes	No	No	No	No	Yes	Yes	Yes
World Health Organisation	Yes	Yes	Yes	No	No	No	Yes	Yes	Yes
PRIVATE DONOR									
Bill & Melinda Gates Foundation	Yes	No	Yes	No	No	No	No	No	No

2.3. Classifying bilateral and multilateral funding

Donor-specific estimates were substantially affected by how each approach categorized donor countries' funding for multilaterals institutions (e.g. UN agencies, the World Bank, GAVI, and the European Union). Donor countries provide aid directly to recipient countries, which is known as "bilateral aid", and to multilateral institutions, which they do in two ways. First, they fund multilaterals' core budgets, which cover administration and activities directed by the multilateral. Second, they provide "earmarked" funds, which allow the donor country to retain control over the recipient country and purpose of the funds.

For each bilateral donor, IHME provided two estimates: 1) for the donor country as the "source" of funds, which included bilateral aid and both types of funding for multilaterals; 2) for the donor country as the "channel" of funds, which included only bilateral aid.

Muskoka, Countdown, and the policy marker estimated donor funding using the same approach as the CRS. They included each country's bilateral disbursements and its earmarked funding disbursed through multilaterals, but excluded its core funding to multilaterals, which was reported separately. These estimates by donor were therefore not directly comparable with IHME's estimates for each country as "source" or "channel" of funds.

The Muskoka approach also generated an additional set of estimates for each donor country, which included its bilateral aid, earmarked funding through multilaterals, and also its core contributions to 10 multilaterals (which did not include the EU).[6] Like IHME's estimates by "source", these additional Muskoka estimates included core contributions to multilaterals as well as earmarked funding for multilaterals and direct bilateral disbursements. For the 23 bilateral donors on which we focus, core contributions to these 10 multilaterals constituted between 18% (USA) and 71% (Finland) of their aid for RMNCH based on the Muskoka approach (Supplementary Table S4). Neither Countdown nor the policy marker credited bilateral donors for their core contributions to multilaterals in any estimates.

The Muskoka approach was originally developed to identify the value of G8 members' aid for RMNCH; the original intention was not to estimate aid from all donors or to examine the value of aid received by individual recipient countries. In this original approach, a separate imputed percentage for each CRS purpose code was applied to each G8 member's bilateral funding and earmarked funding provided through multilaterals. For example, 88.5% of disbursements that donors reported in the "malaria control" purpose code were considered to support RMNCH (Supplementary Table S11). In addition, imputed percentages were applied to each G8 member's core contributions to 10 multilateral institutions. For example, 55% of each donor country's core contributions to UNICEF were counted towards that country's RMNCH funding estimates (Supplementary Table S5). A different institution-based imputed percentage was applied to contributions to each multilateral. The G8 identified these institution-based imputed percentages by asking each multilateral to indicate the proportion of its own expenditure which supported RMNCH in 2009.

In the Muskoka approach, the value of multilateral aid thought to benefit RMNCH and attributed to each bilateral donor is calculated by applying the "institution-based imputed percentages" (Supplementary Table S5) to each donor's core contributions to multilaterals. We obtained data where possible on each of the 24 donors' core contributions to multilaterals from the OECD's DAC2a table (AfDF, IDB Special Fund, UNICEF, IDA, WFP) and multiplied these values by the relevant institution-based imputed percentage. For other multilaterals (AsDF, IBRD, GAVI, Global Fund, UNFPA, WHO), we obtained data from the OECD's table, "Imputed multilateral contributions to the Health sector" (January 2017 version). Of these multilateral institutions, four (WHO, GAVI, Global Fund, UNFPA) only operate within the health sector, meaning that their health sector and all sector disbursements are the same, and so we multiplied contributions to these donors by the relevant institution-based imputed percentage. For the remaining multilaterals (AsDF, IBRD), we estimated each bilateral's total core contributions each year by combining the OECD's estimate of the proportion of each of their expenditure that was in the health sector in 2015 (the only year for which this value was reported) with the OECD's estimates of their core contribution to the health sector in each year. We then multiplied this estimate of total core contributions by the relevant institution-based imputed percentages.

When PMNCH implemented the Muskoka methods, it sought to estimate the total value of aid for RMNCH, rather than to estimate the value of aid provided by individual donors. PMNCH therefore applied Muskoka's purpose-code-based "imputed percentages" to all donors to generate its global estimates. For global and recipient-specific estimates, we replicated PMNCH's approach of applying purpose-code-based imputed percentages to the disbursements from all donors in the CRS. When we generated donor-specific estimates, which PMNCH did not publish, we began by applying this same approach. We then created an additional estimate, which also included the value of core contributions to multilaterals based on the G8's original approach.

Supplementary Table S4 Muskoka methodology: Effects of including bilateral donors' core contributions to multilaterals in estimates of aid for RMNCH, 2002-15

	Cumulative aid for RMNCH (constant 2015 USD, millions)		Donor's multilateral aid as % of its bi+multi aid for RMNCH (row %)	Share of 24 donors' aid for RMNCH (column %)	
Bilateral donor	Bilateral	Multilateral		Bilateral	Bi+Multilateral
Australia	1,695	825	33%	2%	2%
Austria	72	163	69%	0%	0%
Belgium	734	419	36%	1%	1%
Canada	2,961	1,662	36%	4%	4%
Denmark	665	1,033	61%	1%	1%
EU Institutions	3,631	191	5%	5%	3%
Finland	227	559	71%	0%	1%
France	1,464	2,738	65%	2%	4%
Germany	2,700	2,333	46%	3%	4%
Greece	67	20	23%	0%	0%
Ireland	663	277	29%	1%	1%
Italy	413	860	68%	1%	1%
Japan	2,493	2,508	50%	3%	4%
Korea	531	167	24%	1%	1%
Luxembourg	259	132	34%	0%	0%
Netherlands	2,161	2,178	50%	3%	4%
New Zealand	151	101	40%	0%	0%
Norway	1,349	2,873	68%	2%	4%
Portugal	69	37	35%	0%	0%
Spain	1,162	615	35%	2%	2%
Sweden	1,409	2,194	61%	2%	3%
Switzerland	545	708	57%	1%	1%
United Kingdom	9,464	5,296	36%	12%	13%
United States	42,384	9,505	18%	55%	45%

To explore the impact of the different ways PMNCH and the G8 assessed multilateral funding under "the Muskoka method", we compared the proportion of multilateral aid in the CRS that the purpose-code-based and institution-based imputed percentages classified as supporting RMNCH (Supplementary Table S5). We found that the purpose-code-based and institution-based imputed percentages produced dramatically different estimates of support for RMNCH for some multilaterals. For example, the institution-based-imputed percentage indicated that 100% of GAVI funding should be considered to support RMNCH, so the G8 approach and the Muskoka estimates we report by donor credited donor countries for 100% of their core contributions to GAVI. When GAVI reported its disbursements to the CRS, however, it (appropriately) reported that most of its disbursements fell in the "basic health care" purpose code. This meant that when the purpose-code-based imputed percentages were applied to all funding in the CRS to generate the global and recipient-specific Muskoka estimates we report, only 37% of GAVI funding was included in the Muskoka RMNCH estimates. As GAVI is such a large funder of RMNCH, including only 37% of GAVI funding in Muskoka's global RMNCH estimates made them at least $800m per year lower for 2013-15 than they would have been had 100% of GAVI funding been included. The two approaches also included quite different proportions of funding from Unicef: 55% using the institution-based percentages and an average of 10% using the purpose-code-based imputed percentages. The two approaches included more similar proportions of the Global Fund's disbursements: 46% using the institution-based percentages and an average of 54% using the purpose-code-based imputed percentages (Supplementary Table S5).

Supplementary Table S5 Comparison of techniques within the Muskoka method for identifying multilateral aid for RMNCH

Multilateral institution	A: Institution-based imputed %	B: Purpose-code-based imputed %															% of multilateral aid for RMNCH (2002-15) that is from this donor (column %)
	% of each donor's disbursements counted in RMNCH estimate (all years)	% of each donor's disbursements counted in RMNCH estimate															
		Mean, 2002-15*	2002	2003	2004	2005	2006	2007	2008	2009	2010	2011	2012	2013	2014	2015	
African Development Bank	Not included	0.0%	0.0%	0.0%	0.0%	0.0%	0.0%	0.0%	0.0%	0.0%	0.0%	0.2%	0.1%	0.2%	0.2%	0.1%	0.0%
African Development Fund	3%	2.0%	3.4%	2.9%	3.0%	3.2%	0.4%	3.2%	4.0%	2.0%	1.9%	2.4%	1.9%	1.8%	3.2%	2.9%	1.7%
Arab Bank for Economic Development in Africa	Not included	2.8%	No data	No data	No data	No data	No data	No data	No data	No data	No data	3.9%	2.0%	2.2%	2.5%	3.9%	0.0%
Arab Fund (AFESD)	Not included	0.1%	No data	No data	No data	No data	No data	No data	0.2%	0.1%	0.0%	0.2%	0.2%	0.1%	0.3%	0.2%	0.0%
AsDB Special Funds	2%	2.5%	No data	No data	No data	No data	No data	No data	No data	No data	5.1%	2.3%	2.2%	2.0%	1.9%	2.0%	0.8%
Climate Investment Funds	Not included	0.0%	No data	No data	No data	No data	No data	No data	No data	No data	No data	No data	No data	0.0%	0.0%	0.0%	0.0%
Council of Europe Development Bank	Not included	2.3%	No data	No data	No data	No data	No data	No data	No data	No data	2.6%	1.1%	2.6%	2.8%	4.2%	0.9%	0.0%
EU Institutions	Not included	2.5%	3.5%	3.4%	4.2%	2.6%	3.2%	3.0%	2.9%	2.5%	2.3%	2.0%	1.9%	2.2%	2.4%	2.3%	9.7%
Food and Agriculture Organisation	Not included	5.1%	No data	No data	No data	No data	No data	No data	No data	No data	No data	No data	No data	5.1%	No data	No data	0.1%
Global Alliance for Vaccines and Immunization	100%	37.1%	No data	No data	No data	No data	No data	37.8%	37.6%	35.2%	36.3%	36.5%	37.3%	37.0%	37.2%	37.4%	8.7%
Global Environment Facility	Not included	0.0%	0.0%	0.0%	0.0%	0.0%	0.0%	0.0%	0.0%	0.0%	0.0%	0.0%	0.0%	0.0%	0.0%	0.0%	0.0%
Global Fund	46%	53.8%	No data	50.9%	51.3%	55.8%	56.0%	51.2%	52.5%	59.6%	54.5%	51.8%	54.5%	51.7%	54.0%	53.5%	38.3%
IDB Special Fund	1%	1.2%	No data	No data	No data	No data	No data	No data	No data	1.0%	0.7%	1.9%	0.8%	0.7%	1.5%	1.6%	0.4%
IMF (Concessional Trust Funds)	Not included	2.8%	3.4%	3.1%	3.3%	2.3%	0.6%	3.9%	3.9%	3.8%	1.8%	4.0%	3.8%	4.0%	4.0%	3.7%	1.8%
Inter-American Development Bank	Not included	1.8%	No data	No data	No data	No data	No data	No data	No data	No data	No data	No data	No data	No data	No data	1.8%	0.0%
International Development Association	5%	4.3%	6.4%	6.0%	6.5%	7.3%	1.4%	5.0%	4.5%	4.9%	4.9%	5.1%	4.6%	4.5%	3.8%	4.4%	19.8%
Nordic Development Fund	Not included	0.0%	No data	No data	No data	No data	No data	No data	No data	0.0%	0.0%	0.0%	0.0%	0.0%	0.0%	0.2%	0.0%
OPEC Fund for International Development	Not included	3.6%	No data	No data	No data	No data	No data	No data	No data	2.3%	3.4%	4.9%	5.3%	3.9%	3.7%	2.4%	0.2%
UN Peacebuilding Fund	Not included	0.6%	No data	No data	No data	No data	No data	0.0%	0.0%	0.4%	0.4%	0.0%	1.8%	0.0%	1.2%	0.7%	0.0%
UNAIDS	Not included	34.4%	46.1%	46.1%	46.1%	46.0%	46.0%	31.7%	31.2%	33.3%	30.8%	31.4%	33.8%	30.8%	19.5%	29.9%	2.4%
UNDP	Not included	1.8%	No data	No data	1.9%	2.0%	2.2%	1.5%	1.4%	3.0%	2.2%	1.6%	2.3%	1.5%	1.0%	0.8%	0.3%
UNECE	Not included	0.8%	No data	No data	No data	No data	No data	No data	1.2%	0.6%	0.3%	0.0%	1.1%	1.3%	0.8%	1.1%	0.0%
UNFPA	67%	68.2%	85.0%	77.9%	75.6%	74.5%	72.8%	72.8%	70.6%	66.8%	68.4%	74.5%	58.7%	59.3%	58.4%	43.1%	7.4%
UNICEF	55%	10.1%	11.3%	10.6%	10.7%	11.6%	13.2%	9.8%	9.4%	10.2%	10.8%	10.8%	8.4%	9.2%	9.6%	8.9%	3.6%
UNRWA	Not included	7.1%	No data	No data	No data	7.3%	7.5%	8.2%	7.2%	7.4%	7.2%	6.7%	6.8%	6.7%	6.1%	7.8%	1.1%
WFP	15%	5.8%	No data	No data	No data	No data	No data	No data	0.0%	11.8%	12.8%	3.6%	3.6%	6.2%	6.0%	4.5%	0.4%
World Health Organisation	22%	41.1%	No data	No data	No data	No data	No data	No data	No data	42.0%	42.9%	43.2%	41.3%	42.0%	40.5%	38.0%	3.3%
IBRD	5%	NA	No data	No data	No data	No data	No data	No data	No data	No data	No data	No data	No data	No data	No data	No data	NA
Other World Bank (AMCs)	5%	NA	No data	No data	No data	No data	No data	No data	No data	No data	No data	No data	No data	No data	No data	No data	NA
IDA-MDRI	5%	NA	No data	No data	No data	No data	No data	No data	No data	No data	No data	No data	No data	No data	No data	No data	NA

** The mean proportion of each multilateral donor's disbursements counted towards RMNCH over the period 2002-2015 is calculated as the sum of disbursements counted towards RMNCH estimates over the period divided by the total disbursements made by that donor over period.*

2.4. Adjustments for inflation and exchange rates

To compare estimates across donors and years, each currency-year of expenditure must be adjusted to its equivalent in a single currency and year using “deflators”. Supplementary Table S6 compares two sets of deflators: 1) The OECD's CRS deflators, which include an adjustment for inflation in the donor country and variation in each year's average exchange rates with the United States dollar (USD) and were used by Countdown, Muskoka, and the OECD’s policy marker and 2) the deflators generated based on IHME's methods, which first adjust each year's expenditure into USD, and then account for inflation using GDP estimates for the United States.

In Supplementary Table S7, we show how much higher or lower IHME's estimates would have been had the relevant DAC deflator been used instead. Results for seven major donors are presented as illustrative examples. The most extreme differences between deflators occur in periods in which a donor’s rate of inflation differed substantially from that of the United States. Differences in the choice of deflators thus have the largest impact on estimates for donors whose economic cycles and growth rates have differed most substantially from those of the United States, notably Japan and Australia.

Supplementary Table S6 Magnitude of differences in IHME and OECD deflators

A value in a given year's current dollars can be converted to constant 2015 dollars by dividing the current dollar value by the deflator for that year. This is the traditional manner for presenting deflators, which reflect changes in the relative price level over time.

		Australia		Canada		France		Germany		Japan		United Kingdom		United States	
	IHME	Min	-31%	Min	-23%	Min	-29%	Min	-36%	Min	-57%	Min	-21%	Min	-9%
	deflator	Max	61%	Max	23%	Max	13%	Max	11%	Max	0%	Max	10%	Max	1%
Year	used for all currencies	OECD DAC deflator	% difference in deflator	OECD DAC deflator	% difference in deflator	OECD DAC deflator	% difference in deflator	OECD DAC deflator	% difference in deflator	OECD DAC deflator	% difference in deflator	OECD DAC deflator	% difference in deflator	OECD DAC deflator	% difference in deflator
1990	0.55	0.57	-3%	0.67	-18%	0.76	-28%	0.76	-28%	0.94	-41%	0.63	-13%	0.61	-9%
1991	0.57	0.58	-1%	0.71	-19%	0.76	-24%	0.76	-24%	1.03	-44%	0.67	-14%	0.63	-9%
1992	0.59	0.55	7%	0.68	-13%	0.82	-28%	0.85	-30%	1.11	-47%	0.69	-14%	0.64	-8%
1993	0.61	0.52	18%	0.65	-6%	0.78	-22%	0.83	-27%	1.27	-52%	0.60	1%	0.66	-7%
1994	0.63	0.56	11%	0.62	1%	0.80	-22%	0.87	-28%	1.39	-55%	0.62	1%	0.67	-7%
1995	0.64	0.58	10%	0.63	2%	0.90	-29%	1.00	-36%	1.50	-57%	0.66	-2%	0.69	-6%
1996	0.66	0.63	5%	0.64	3%	0.89	-26%	0.96	-31%	1.29	-49%	0.68	-2%	0.70	-5%
1997	0.68	0.61	12%	0.64	6%	0.79	-14%	0.84	-19%	1.16	-42%	0.73	-7%	0.71	-5%
1998	0.69	0.51	34%	0.60	15%	0.79	-13%	0.83	-17%	1.08	-36%	0.75	-8%	0.72	-4%
1999	0.70	0.54	31%	0.61	16%	0.76	-7%	0.80	-12%	1.22	-42%	0.74	-4%	0.73	-4%
2000	0.73	0.50	45%	0.63	15%	0.67	9%	0.69	6%	1.27	-43%	0.70	3%	0.75	-3%
2001	0.75	0.46	61%	0.62	21%	0.66	13%	0.68	11%	1.12	-33%	0.68	10%	0.76	-2%
2002	0.76	0.50	51%	0.62	23%	0.71	7%	0.72	5%	1.07	-29%	0.72	5%	0.77	-2%
2003	0.78	0.62	26%	0.72	8%	0.87	-10%	0.87	-11%	1.13	-32%	0.81	-4%	0.79	-2%
2004	0.80	0.73	10%	0.80	0%	0.97	-18%	0.97	-18%	1.20	-33%	0.93	-15%	0.81	-2%
2005	0.82	0.78	5%	0.88	-6%	0.99	-16%	0.98	-16%	1.16	-29%	0.95	-14%	0.84	-2%
2006	0.85	0.81	4%	0.97	-12%	1.02	-16%	0.99	-14%	1.09	-22%	0.99	-14%	0.86	-1%
2007	0.87	0.95	-7%	1.05	-17%	1.14	-23%	1.10	-20%	1.06	-18%	1.11	-21%	0.89	-1%
2008	0.91	0.99	-8%	1.09	-17%	1.23	-26%	1.17	-22%	1.20	-24%	1.03	-12%	0.90	1%
2009	0.91	0.94	-4%	1.01	-10%	1.19	-24%	1.15	-21%	1.32	-31%	0.91	-1%	0.91	-1%
2010	0.92	1.16	-21%	1.15	-20%	1.14	-19%	1.10	-16%	1.37	-33%	0.93	-1%	0.92	0%
2011	0.95	1.37	-31%	1.24	-23%	1.21	-22%	1.17	-19%	1.48	-36%	0.98	-4%	0.94	1%
2012	0.97	1.37	-29%	1.24	-22%	1.13	-14%	1.09	-11%	1.47	-34%	0.99	-2%	0.96	1%
2013	0.98	1.29	-24%	1.22	-19%	1.18	-17%	1.15	-15%	1.19	-18%	1.00	-1%	0.97	1%
2014	1.00	1.21	-17%	1.16	-14%	1.18	-16%	1.17	-15%	1.12	-11%	1.07	-6%	0.99	1%
2015	1.00	1.00	0%	1.00	0%	1.00	0%	1.00	0%	1.00	0%	1.00	0%	1.00	0%

Supplementary Table S7 Effect of choice of deflators on IHME estimates

This table presents multipliers, which are the reciprocal of the deflators presented in Supplementary Table S6. A value in a given year's current dollars can be converted to constant 2015 dollars by multiplying the current dollar value by the multiplier for that year. The "% difference in estimates" indicates how much higher or lower IHME's estimates would have been had the DAC deflator been used instead.

		Australia		Canada		France		Germany		Japan		United Kingdom		United States	
		Min	-38%	Min	-19%	Min	-12%	Min	-10%	Min	0%	Min	-9%	Min	-1%
		Max	44%	Max	30%	Max	41%	Max	56%	Max	133%	Max	27%	Max	10%
Year	IHME multiplier used for all currencies	OECD DAC multiplier	% difference in estimates	OECD DAC multiplier	% difference in estimates	OECD DAC multiplier	% difference in estimates	OECD DAC multiplier	% difference in estimates	OECD DAC multiplier	% difference in estimates	OECD DAC multiplier	% difference in estimates	OECD DAC multiplier	% difference in estimates
1990	1.81	1.76	3%	1.49	22%	1.31	38%	1.31	38%	1.07	70%	1.59	14%	1.64	10%
1991	1.74	1.73	1%	1.42	23%	1.32	32%	1.32	32%	0.97	80%	1.50	16%	1.59	9%
1992	1.69	1.81	-7%	1.47	15%	1.22	39%	1.18	43%	0.90	88%	1.46	16%	1.56	9%
1993	1.64	1.94	-15%	1.55	6%	1.28	28%	1.20	37%	0.78	109%	1.66	-1%	1.52	8%
1994	1.60	1.78	-10%	1.62	-1%	1.24	28%	1.15	39%	0.72	122%	1.61	-1%	1.49	7%
1995	1.56	1.71	-9%	1.59	-2%	1.11	41%	1.00	56%	0.67	133%	1.52	2%	1.46	7%
1996	1.51	1.59	-5%	1.55	-3%	1.12	35%	1.04	45%	0.78	95%	1.48	2%	1.43	6%
1997	1.48	1.65	-10%	1.56	-5%	1.27	17%	1.20	23%	0.86	72%	1.38	7%	1.41	5%
1998	1.45	1.94	-25%	1.67	-13%	1.27	15%	1.21	20%	0.93	56%	1.34	8%	1.39	4%
1999	1.42	1.86	-24%	1.64	-13%	1.32	8%	1.26	13%	0.82	74%	1.36	5%	1.37	4%
2000	1.38	2.00	-31%	1.58	-13%	1.50	-8%	1.46	-6%	0.78	75%	1.42	-3%	1.34	3%
2001	1.34	2.16	-38%	1.62	-17%	1.52	-12%	1.48	-10%	0.90	50%	1.48	-9%	1.31	2%
2002	1.32	1.99	-34%	1.62	-19%	1.41	-7%	1.39	-5%	0.94	41%	1.38	-5%	1.29	2%
2003	1.29	1.62	-20%	1.40	-8%	1.16	11%	1.15	12%	0.88	46%	1.24	4%	1.27	2%
2004	1.25	1.38	-9%	1.26	0%	1.03	21%	1.03	22%	0.83	50%	1.07	17%	1.23	2%
2005	1.21	1.27	-5%	1.13	7%	1.01	20%	1.02	19%	0.86	41%	1.05	16%	1.19	2%
2006	1.18	1.23	-4%	1.03	14%	0.98	20%	1.01	16%	0.92	28%	1.01	17%	1.16	2%
2007	1.14	1.06	8%	0.95	20%	0.88	30%	0.91	25%	0.94	22%	0.90	27%	1.13	1%
2008	1.10	1.01	9%	0.91	20%	0.81	35%	0.86	28%	0.84	32%	0.97	14%	1.11	-1%
2009	1.10	1.06	4%	0.99	11%	0.84	31%	0.87	27%	0.76	46%	1.10	1%	1.10	1%
2010	1.09	0.86	27%	0.87	25%	0.88	24%	0.91	19%	0.73	49%	1.08	1%	1.08	0%
2011	1.05	0.73	44%	0.81	30%	0.83	27%	0.86	23%	0.67	56%	1.02	4%	1.06	-1%
2012	1.03	0.73	41%	0.81	28%	0.88	17%	0.91	13%	0.68	51%	1.01	2%	1.04	-1%
2013	1.02	0.78	31%	0.82	24%	0.85	20%	0.87	17%	0.84	21%	1.01	1%	1.03	-1%
2014	1.00	0.83	21%	0.86	16%	0.84	18%	0.85	17%	0.89	12%	0.94	7%	1.01	-1%
2015	1.00	1.00	0%	1.00	0%	1.00	0%	1.00	0%	1.00	0%	1.00	0%	1.00	0%

2.5. Recipients

The 75 Countdown to 2015 priority recipients are listed in *Supplementary Table S8.*

Supplementary Table S8 The 75 Countdown to 2015 priority recipients

Afghanistan	Gabon	Nigeria
Angola	Gambia	Pakistan
Azerbaijan	Ghana	Papua New Guinea
Bangladesh	Guatemala	Peru
Benin	Guinea	Philippines
Bolivia	Guinea-Bissau	Rwanda
Botswana	Haiti	Sao Tome and Principe
Brazil	India	Senegal
Burkina Faso	Indonesia	Sierra Leone
Burundi	Iraq	Solomon Islands
Cambodia	Kenya	Somalia
Cameroon	Kyrgyz Republic	South Africa
Central African Republic	Lao People's Democratic Republic	South Sudan
Chad	Lesotho	Sudan
China (People's Republic of)	Liberia	Swaziland
Comoros	Madagascar	Tajikistan
Congo, Rep.	Malawi	Tanzania
Cote d'Ivoire	Mali	Togo
Democratic People's Republic of Korea	Mauritania	Turkmenistan
Democratic Republic of the Congo	Mexico	Uganda
Djibouti	Morocco	Uzbekistan
Egypt	Mozambique	Vietnam
Equatorial Guinea	Myanmar	Yemen
Eritrea	Nepal	Zambia
Ethiopia	Niger	Zimbabwe

We compared the proportion of the estimates of aid for RMNCH within each method's dataset that was disbursed to specified countries, regions, global, and unspecified recipients (*Supplementary Figure S5*). In 2013, 5% to 6% of Muskoka and Countdown estimates were disbursed to regions, and 14% to 22% were disbursed to "unspecified" recipients. In generating estimates of the value of aid for RMNCH for individual recipient countries and the 75 priority countries as a group, Countdown and Muskoka attributed shares of regional and unspecified funding to individual recipient countries.

IHME included regional funding in its estimates for individual recipient countries and the 75 priority countries as a group, but excluded global and unspecified funding. The policy marker estimates excluded regional and unspecified funding from estimates for individual recipient countries and the 75 priority countries as a group. These exclusions reduced the policy marker's country-specific estimates by ~30% and IHME's by ~55% on average. On average, IHME classified 45% of the funding in its dataset (1990-2014) as being disbursed to a specified recipient country, 12% as "global", and 43% as "unallocated/unspecified". Whereas WHO, UNICEF, and UNFPA provided country-specific recipient data for 62% of the funding Muskoka classified as MNCH in 2013, in IHME's dataset, the recipient of all funding flowing through these three institutions (which together accounted for 27% of IHME's MNCH estimate) was "unallocated/unspecified"; this discrepancy accounts to some extent for the lower overall proportion of recipient-country-specific data in IHME's MNCH estimates.

Supplementary Figure S5 Disbursements to specified countries, regions, global, and unspecified recipients

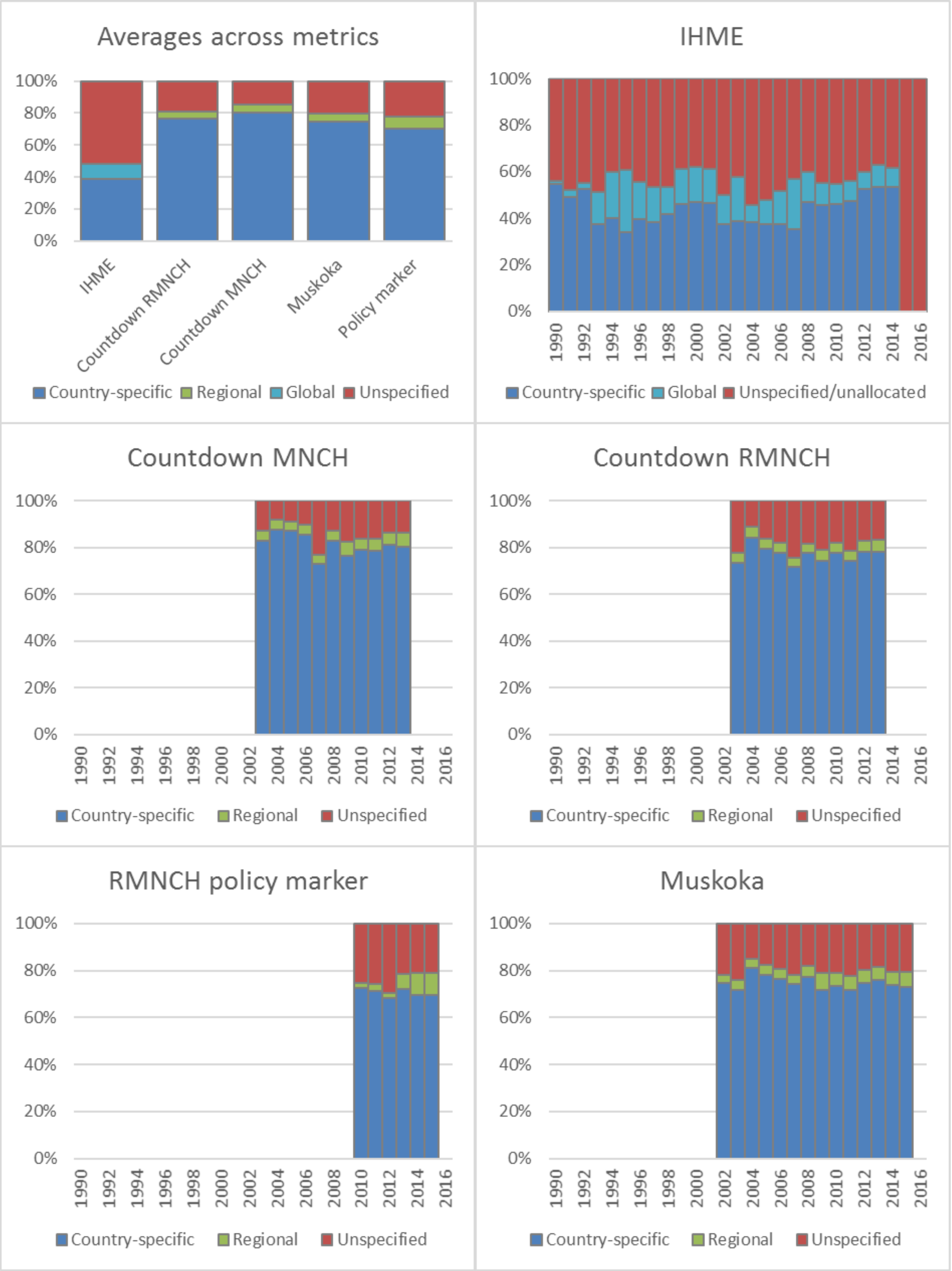

2.7. Adjustments for underreporting and reporting lags

Only IHME substantially adjusted donors' data for underreporting and reporting lags. Countdown included a minor adjustment (described below), while Muskoka and policy marker estimates were not adjusted for underreporting.

The OECD discourages use of CRS disbursement data for the 1990s and early 2000s because donors severely underreported their disbursements.[9] For these years, Muskoka and Countdown therefore did not report estimates and policy marker estimates are not available. To estimate disbursements in these years with very incomplete disbursement data (largely before 2000-2005, depending on donor), IHME used CRS commitment data, which were more complete, to model bilateral and EU disbursements and to categorize them as focused on MNCH or other areas from 1990 onwards.[10] As detailed commitment data were only available for <50% and often <10% of the value of health sector commitments reported in aggregate, IHME inflated their value to match aggregate commitments. This assumed that available data was representative of missing data.

For the decade until 2014, IHME's MNCH estimates reflected disbursement data with minor adjustments to match reported commitments, although the exact magnitude of these adjustments is unclear. Countdown's only adjustment for underreporting consisted of obtaining GAVI disbursement data for 2003-6 directly from GAVI, which amounted to $151m to $222m for each of those years.

CRS data is reported by calendar year with substantial delays. Relatively complete data for 2014 became available in January 2016 and was substantially revised in April 2016. IHME addressed these delays by using donor budgets and regression models to forecast "estimated disbursements" for MNCH two calendar years (2015-16) beyond the published CRS disbursement data. This adjustment allowed IHME to report global and donor-specific (but not recipient-specific) estimates for 2015 and 2016. How IHME's projections compared with data reported subsequently is not clear.

Because of differences in the time taken to implement each approach once data are available, the latest Countdown estimates we report are for 2013, while the latest Muskoka and policy marker estimates are for 2015. For PMNCH reports on Muskoka estimates, recent expenditure data were also collected from key donors; they were used to indicate recent trends in the report text but were not included in their figures or reported estimates and are not reported here.

2.8. How aid for RMNCH was distinguished from aid for other purposes

We first present a table comparing how specific services and activities were classified in the purpose code framework of the OECD's Creditor Reporting System (CRS) and by each of the four RMNCH aid tracking approaches (Supplementary Table S9). We then present the composition of Muskoka and Countdown RMNCH estimates by CRS purpose and sector codes, both in total (Supplementary Figure S6) and for each of the 24 recipient countries on which we focus (Supplementary Figure S7, Supplementary Figure S8). These figures illustrate that some of the funding Muskoka and Countdown categorize as supporting RMNCH were coded in purpose codes for reproductive health, family planning, and nutrition, but a substantial proportion of estimates were also comprised of funding in the HIV, malaria, and other health purpose codes, as well as in the humanitarian sector and in other sectors. The composition of RMNCH estimates varied substantially across recipient countries.

To explore the effects of these different definitions and procedures in greater depth, we applied the IHME, Muskoka, and Countdown procedures for distinguishing aid for RMNCH from aid for other purposes in the Countdown dataset, which covers the period 2003-13. This analysis is restricted to aid from the 24 donors whose aid IHME assessed based on CRS data. This analysis holds all other technical choices constant, and therefore illustrates the effects on estimates of differences in allocation procedures alone. Regional and unspecified disbursements are not included in recipient-specific estimates in this analysis. We then present a table (Supplementary Table S11) and various figures (Supplementary Figure S10, Supplementary Figure S11, Supplementary Figure S12, Supplementary Figure S13) showing the results of this analysis within the Countdown dataset.

Supplementary Table S9 Classification of specific services and activities in Creditor Reporting System (CRS) purpose codes and four RMNCH tracking initiatives

The table lists selected potentially relevant activity areas for RMNCH and indicates how each method seeks to categorise funding flows targeting that activity area. For the creditor reporting system, the purpose code(s) under which such an activity should be coded is indicated. For each RMNCH aid tracking method, the degree to which such funding is intended to be coded as RMNCH is indicated. RMNCH=reproductive, maternal, newborn, and child health. IHME=Institute for Health Metrics and Evaluation. OECD=Organisation for Economic Co-operation and Development. *While the guidelines for coding the OECD policy marker did not explicitly exclude general budget support, it is virtually impossible for a general budget support disbursement to meet the minimum threshold (>12.5% of its value benefitting RMNCH) to be coded as benefitting RMNCH.

Service / activity area	OECD's Creditor Reporting System (CRS) purpose codes	Countdown	IHME	Muskoka	OECD RMNCH policy marker
Research	Research is included in the definitions for the "medical research" (12182) and "population policy and administrative management" (13010) purpose codes, but research related to "basic health" has no dedicated purpose code and is spread throughout the "basic health" sector (code 120)	Not RMNCH	Some	Not RMNCH	Some
Maternal health	"Reproductive health" purpose code (13020)	Yes	Yes	Yes	Yes
Nutrition	"Basic nutrition" purpose code (12240)	Yes	Yes	Yes	Yes
Family planning	"Family planning" purpose code (13030)	Yes	Yes	Yes	Yes
Child health	No specific purpose code; infectious disease activities fall under malaria, HIV, tuberculosis, or infectious disease purpose codes; activities at primary or community level fall under "basic health care" (12220); and non-communicable diseases, mental health, and tertiary hospital activities all fall under "medical services" (12191)	Yes	Yes	Yes	Yes
HIV	"Sexually transmitted disease control including HIV/AIDS" purpose code (13040)	Some	Not RMNCH	Some	Some
Sexually-transmitted infections	"Sexually transmitted disease control including HIV/AIDS" purpose code (13040)	Yes	Not RMNCH	Some	Some
Malaria	"Malaria control" purpose code (12262)	Some	Not RMNCH	Some	Some
Vaccine-preventable child illnesses	"Infectious disease control" purpose code (12250)	Yes	Yes	Some	Yes
Other infectious diseases	"Infectious disease control" purpose code (12250)	Some	Not RMNCH	Some	Some
Water and sanitation	"Water and sanitation" sector code (140)	Not RMNCH	Not health	Some	Some
Humanitarian aid	"Humanitarian aid" sector code (720)	Some	Not health	Not RMNCH	Some
General budget support	"General budget support" purpose code (51010)	Some	Not health	Some	Not RMNCH*

Supplementary Figure S6 Muskoka and Countdown RMNCH estimates over time by purpose and sector codes

This figure shows the composition of Muskoka RMNCH and Countdown RMNCH estimates by purpose and sector codes in the Creditor Reporting System. Key purpose codes and sectors are shown individually, and others are grouped.

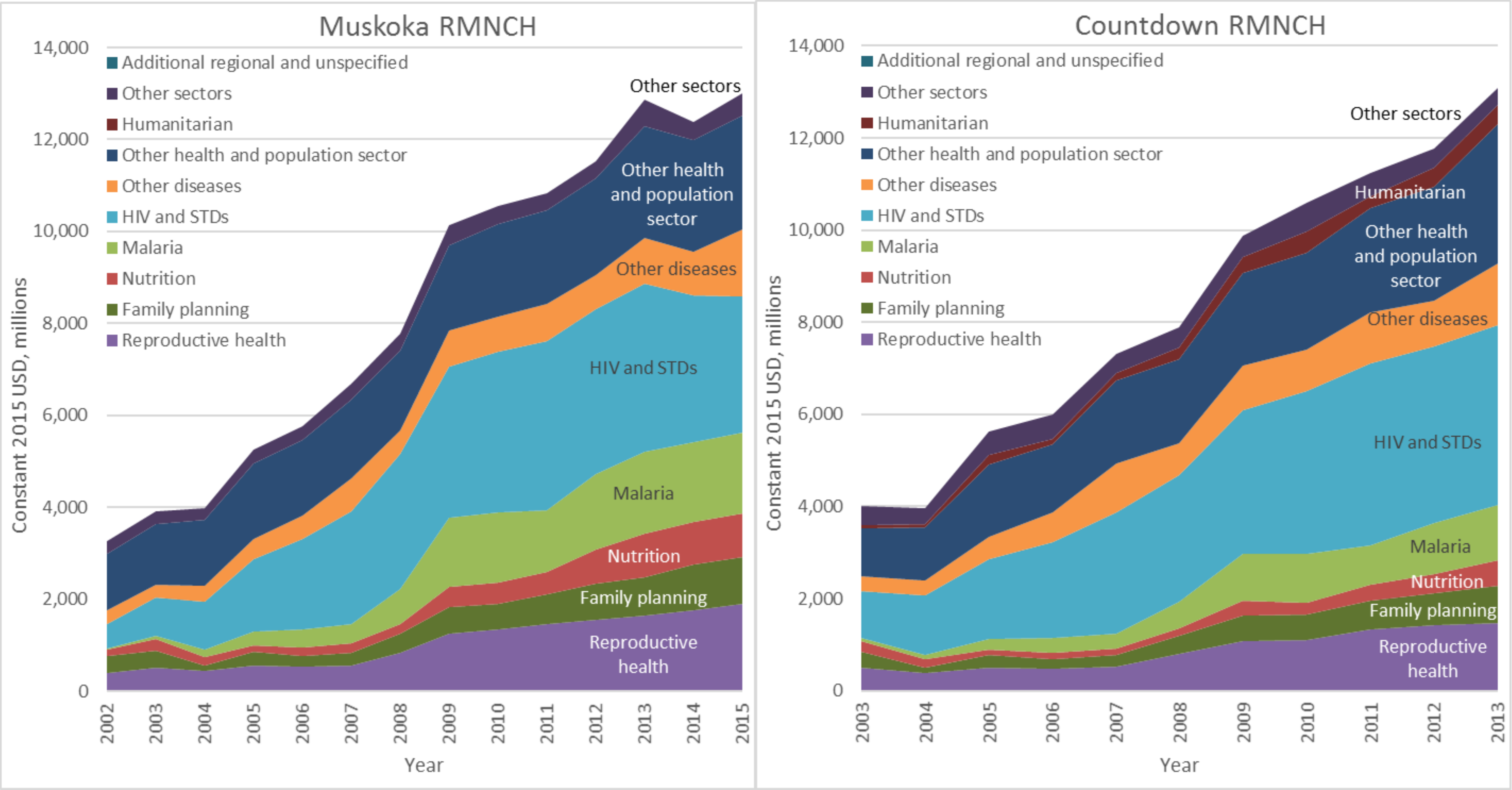

Supplementary Figure S7 Muskoka RMNCH estimates by CRS purpose and sector codes and by recipient country, 2002-15

This figure shows the composition of Muskoka RMNCH estimates by purpose and sector codes in the Creditor Reporting System. Estimates are presented separately for the 24 recipient countries on which we focus throughout the article.

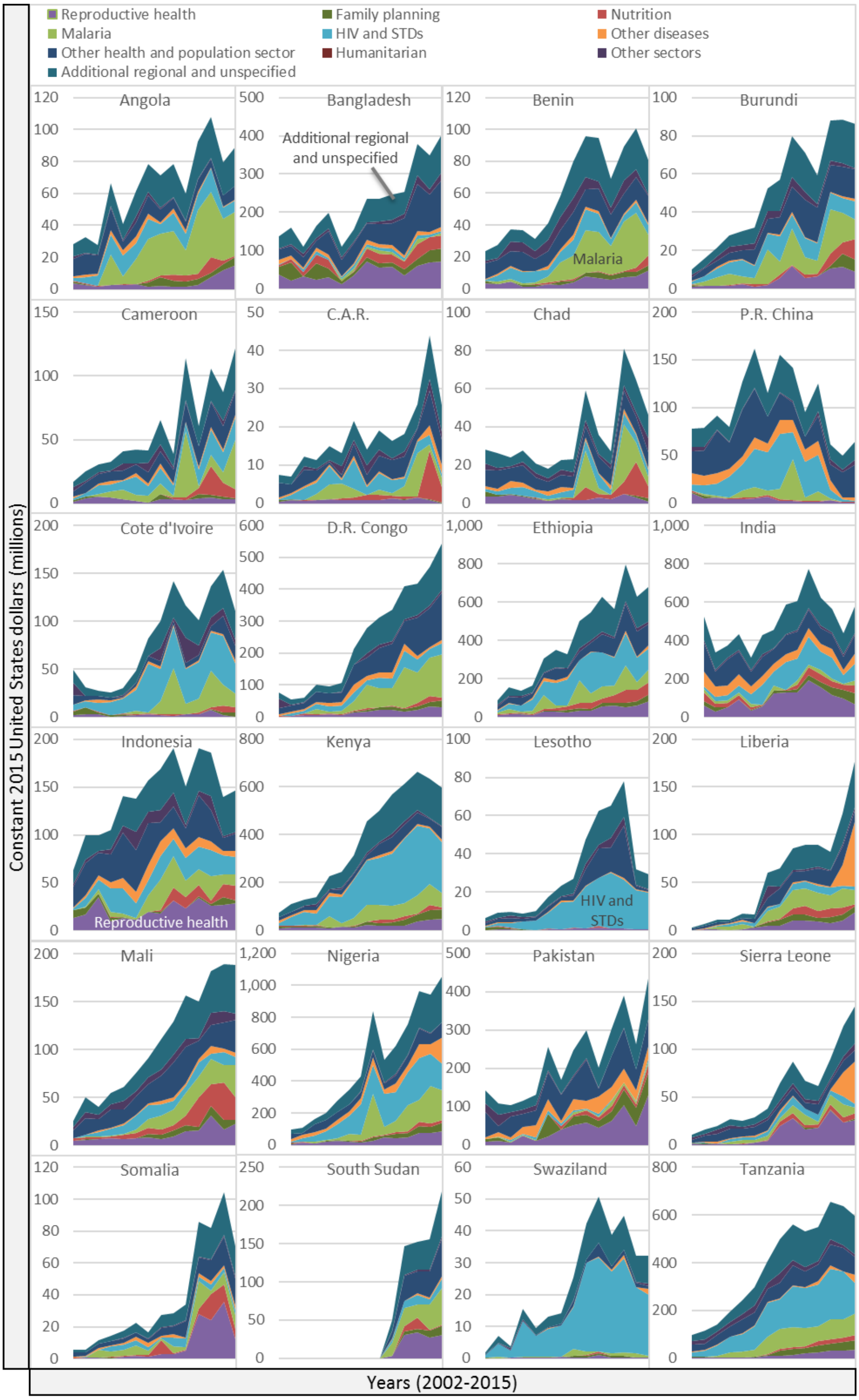

Supplementary Figure S8 Countdown RMNCH estimates by CRS purpose and sector codes and by recipient, 2003-13

This figure shows the composition of Countdown RMNCH estimates by purpose and sector codes in the Creditor Reporting System. Estimates are presented separately for the 24 recipient countries on which we focus throughout the article.

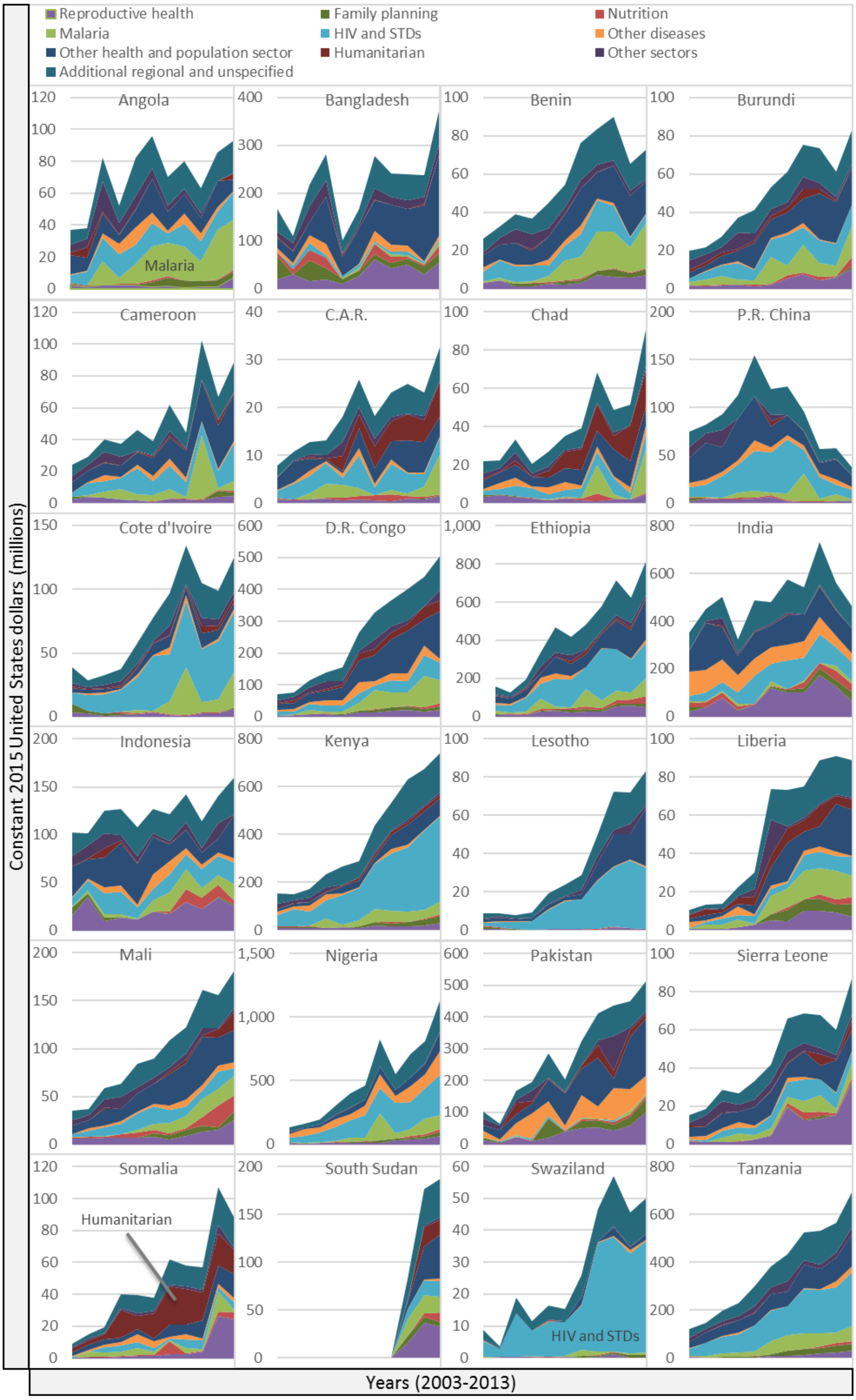

Supplementary Table S10 Comparison of the composition of estimates of aid for RMNCH in 2013 by sector and purpose code

This table reflects the most recent estimates available at the time of our analysis for all donors to all recipients included by each approach. We present key sectors and purpose codes and aggregate all others. *IHME's publicly available database does not include any data on purpose codes, so the proportions presented reflect our recreation of the IHME procedures for allocating aid to MNCH within the Countdown dataset, restricted to funding flows from 24 bilateral donors in 2013, although the total estimate of aid for RMNCH is its published estimate for all donors. *While the OECD's directives to donors for coding the RMNCH policy marker do not explicitly exclude general budget support, they require that at least 12.5% of the value of a given project supports RMNCH for any of the value of that disbursement to be counted towards RMNCH, which effectively excludes support to general government expenditure.

			Composition of each metric's estimate of aid for RMNCH by sector and purpose code									
			Countdown RMNCH		Countdown MNCH		Muskoka RMNCH		OECD RMNCH policy marker		IHME MNCH*	
Sector	Purpose	TOTAL aid for (R)MNCH in 2013 (constant 2015 USD)	$13,088m (100%)		$8,493m (100%)		$12,872m (100%)		$2,006m (100%)		$10,822m (100%)	
120	(All)	HEALTH	42.4%		66.6%		46.4%		31.3%		45.2%	
	12110	Health policy and administrative management		6.1%		9.3%		4.8%		5.8%		3.4%
	12181	Medical education/training		0.3%		0.4%		0.3%		0.3%		0.1%
	12182	Medical research		0.0%		0.0%		0.0%		1.6%		1.1%
	12191	Medical services		1.1%		1.6%		1.6%		1.7%		0.8%
	12220	Basic health care		12.3%		19.5%		8.9%		7.1%		8.4%
	12230	Basic health infrastructure		1.7%		2.7%		1.1%		1.7%		1.0%
	12240	Basic nutrition		3.1%		5.0%		7.3%		6.1%		11.6%
	12250	Infectious disease control		8.9%		14.0%		6.0%		4.1%		17.7%
	12261	Health education		0.3%		0.4%		0.5%		0.5%		0.3%
	12262	Malaria control		7.5%		12.0%		14.0%		1.0%		Excluded
	12263	Tuberculosis control		0.8%		1.1%		1.6%		0.3%		Excluded
	12281	Health personnel development		0.3%		0.5%		0.3%		1.2%		0.6%
130	(All)	POPULATION POLICIES/ PROGRAMMES AND REPROD	49.1%		20.6%		49.1%		50.1%		54.8%	
	13010	Population policy and administrative management		0.5%		0.4%		1.0%		1.5%		1.1%
	13020	Reproductive health care		10.5%		14.4%		12.8%		26.9%		32.9%
	13030	Family planning		5.3%		0.2%		6.5%		16.3%		20.4%
	13040	STD control including HIV/AIDS		32.6%		5.5%		28.4%		4.2%		Excluded
	13081	Personnel development for pop. and repro. health		0.2%		0.2%		0.5%		1.3%		0.5%
720	(All)	Emergency Response	3.0%		4.7%		Excluded		12.6%		Excluded	
	72010	Material relief assistance and services		2.3%		3.7%		Excluded		2.8%		Excluded
	72040	Emergency food aid		0.5%		0.9%		Excluded		3.3%		Excluded
	72050	Relief co-ordination; protection and support services		0.1%		0.2%		Excluded		6.4%		Excluded
110	(All)	EDUCATION	0.2%		0.3%		Excluded		1.3%		Excluded	
140	(All)	WATER AND SANITATION	0.3%		0.4%		1.8%		1.1%		Excluded	
510	51010	General budget support-related aid		1.5%		2.4%		2.7%		Excluded*		Excluded
		Other	3.5%		5.0%		Excluded		3.6%		Excluded	

Supplementary Table S11 Comparison of the composition of estimates of aid for RMNCH by sector and purpose code (replication in Countdown dataset for 24 bilateral donors)

This table reflects our recreation of the Muskoka and IHME methods for apportioning aid to RMNCH within the Countdown dataset, restricted to flows from 24 bilateral donors. We held all other technical choices constant to isolate the effects on estimates of each method's RMNCH definitions and allocation procedures alone. We present the composition of each estimate by sector and activity area (column percentages). We present key sectors and purpose codes and aggregate all others.

			% of disbursements counted towards estimates of aid for RMNCH							
Sector	Purpose	Description	Countdown RMNCH		Countdown MNCH		Muskoka RMNCH		IHME MNCH	
120	(All)	HEALTH	42.65%		41.87%		44.45%		21.67%	
	12110	Health policy and administrative management		33.53%		32.59%		40.00%		8.13%
	12181	Medical education/training		30.57%		29.70%		40.00%		4.04%
	12182	Medical research		0.30%		0.30%		0.00%		10.35%
	12191	Medical services		32.55%		30.87%		40.00%		8.23%
	12220	Basic health care		47.15%		46.47%		40.00%		16.49%
	12230	Basic health infrastructure		39.50%		38.80%		40.00%		6.55%
	12240	Basic nutrition		73.74%		73.71%		100.00%		100.00%
	12250	Infectious disease control		48.94%		48.13%		40.00%		46.08%
	12261	Health education		38.49%		31.67%		40.00%		13.89%
	12262	Malaria control		64.96%		64.96%		88.50%		Excluded
	12263	Tuberculosis control		12.89%		11.57%		18.50%		Excluded
	12281	Health personnel development		44.47%		43.74%		40.00%		25.56%
130	(All)	POPULATION POLICIES/ PROGRAMMES AND REPRODU	57.18%		14.29%		57.02%		21.01%	
	13010	Population policy and administrative management		13.12%		4.79%		40.00%		11.37%
	13020	Reproductive health care		95.23%		80.70%		100.00%		100.00%
	13030	Family planning		96.06%		2.20%		100.00%		100.00%
	13040	STD control including HIV/AIDS		48.94%		4.72%		46.10%		Excluded
	13081	Personnel dev. for pop. and repro. health		86.30%		73.70%		100.00%		62.52%
720	(All)	EMERGENCY RESPONSE	2.72%		2.67%		Excluded		Excluded	
110	(All)	EDUCATION	0.21%		0.19%		Excluded		Excluded	
140	(All)	WATER AND SANITATION	0.50%		0.50%		3.69%		Excluded	
510	51010	General budget support-related aid		2.38%		2.37%		4.00%		Excluded
		All other sectors and purpose codes	0.22%		0.20%		Excluded		Excluded	

Supplementary Figure S9 Countdown vs IHME vs Muskoka to classify aid for RMNCH and other purposes, 2003–13

This Sankey diagram shows how the same funding flows are categorised by Countdown, IHME, and Muskoka. Data in this diagram reflect all official development assistance from 23 longstanding bilateral donors and the EU in the Countdown database, which covers the period 2003–13. The same Countdown data is presented on both the left and right sides of the figure to show all pairwise relationships. IHME=Institute for Health Metrics and Evaluation. RMNCH=reproductive, maternal, newborn, and child health. R*=family planning, sexual health, and sexually transmitted infections, including HIV. MNH=maternal and newborn health.

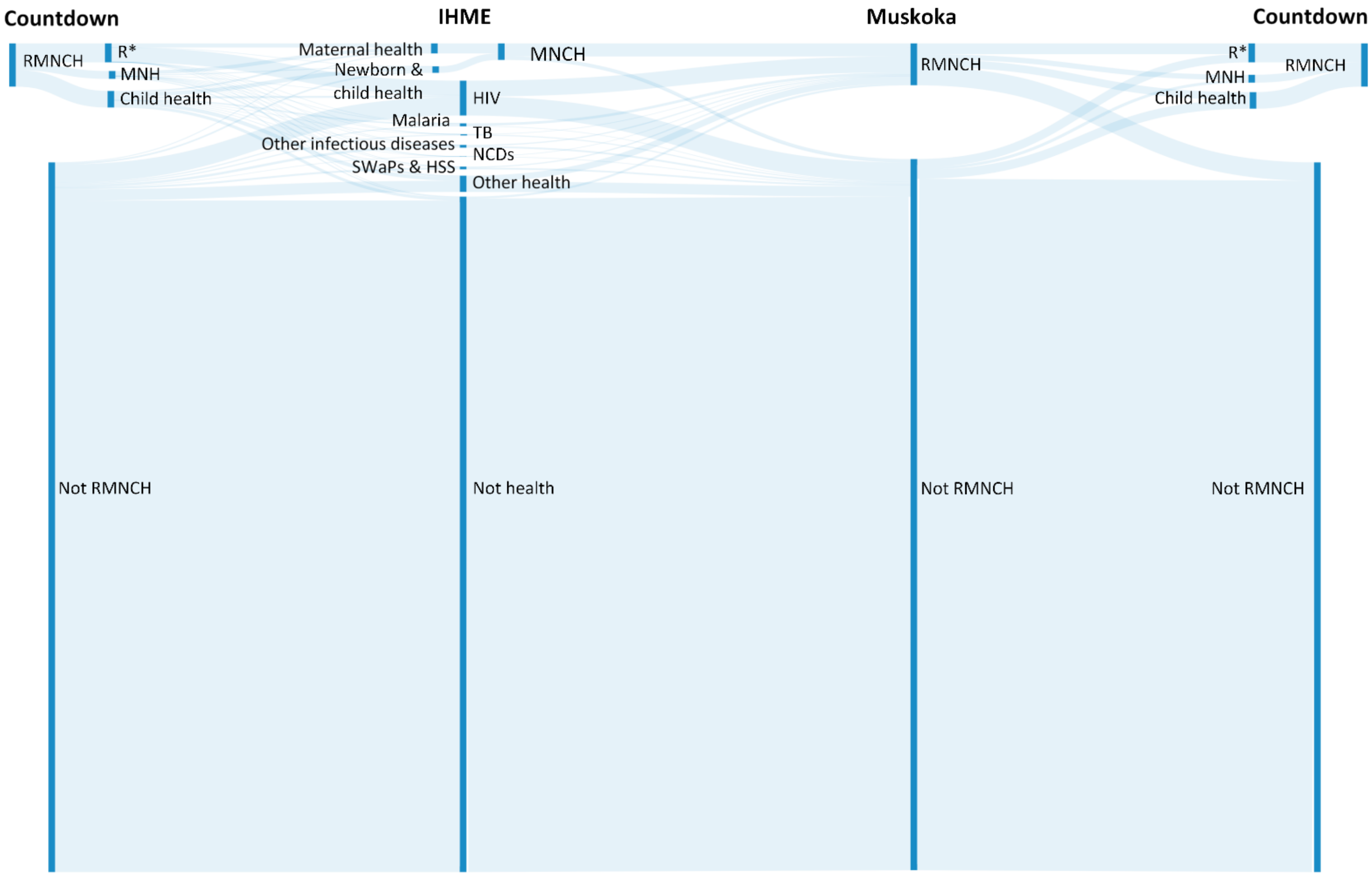

Supplementary Figure S10 Effects of differing RMNCH definitions and allocation procedures on estimates and trends in aid for RMNCH from 24 bilateral donors, 2003-2013

This figure reflects our recreation of the Muskoka and IHME methods for apportioning aid to RMNCH within the Countdown dataset, restricted to flows from 24 bilateral donors. We held all other technical choices constant to isolate the effects on estimates of each method's RMNCH definitions and allocation procedures alone. IHME: Institute for Health Metrics and Evaluation. OECD: Organization for Economic Co-operation and Development.

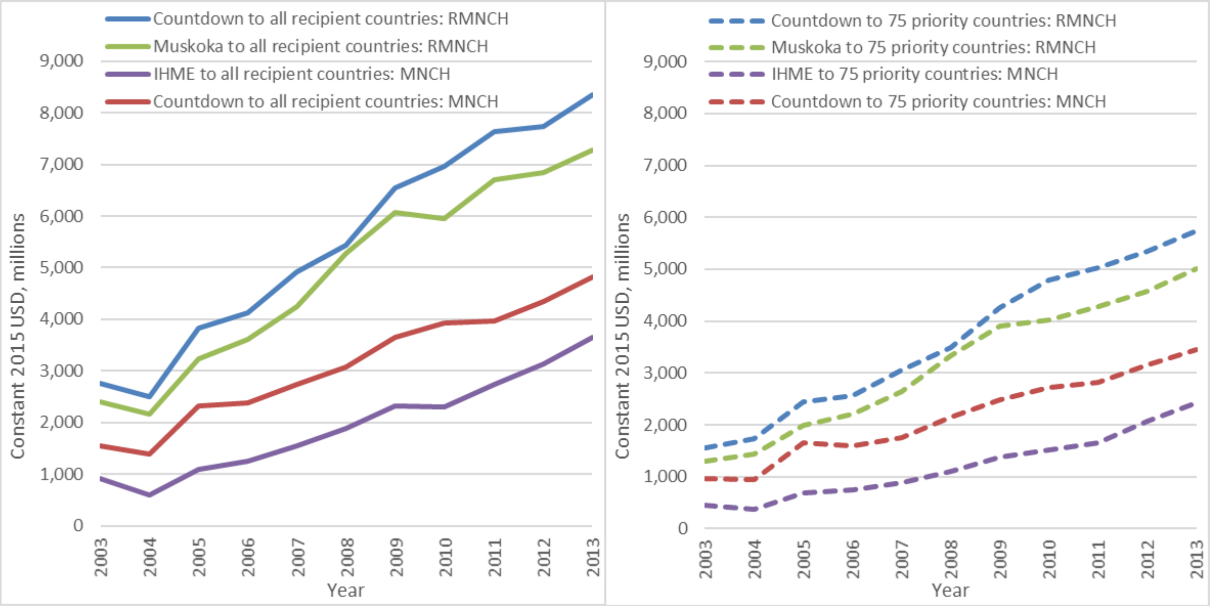

Supplementary Figure S11 Effects of differing RMNCH definitions and allocation procedures on estimates and trends in aid for RMNCH from 24 bilateral donors, 2003-2013

This figure reflects our recreation of Muskoka and IHME's methods for identifying aid for RMNCH within the Countdown dataset, restricted to flows from 24 bilateral donors and disaggregated by donor. We held all other technical choices constant to isolate the effects on estimates of each method's RMNCH definitions and allocation procedures alone. IHME: Institute for Health Metrics and Evaluation. OECD: Organization for Economic Co-operation and Development.

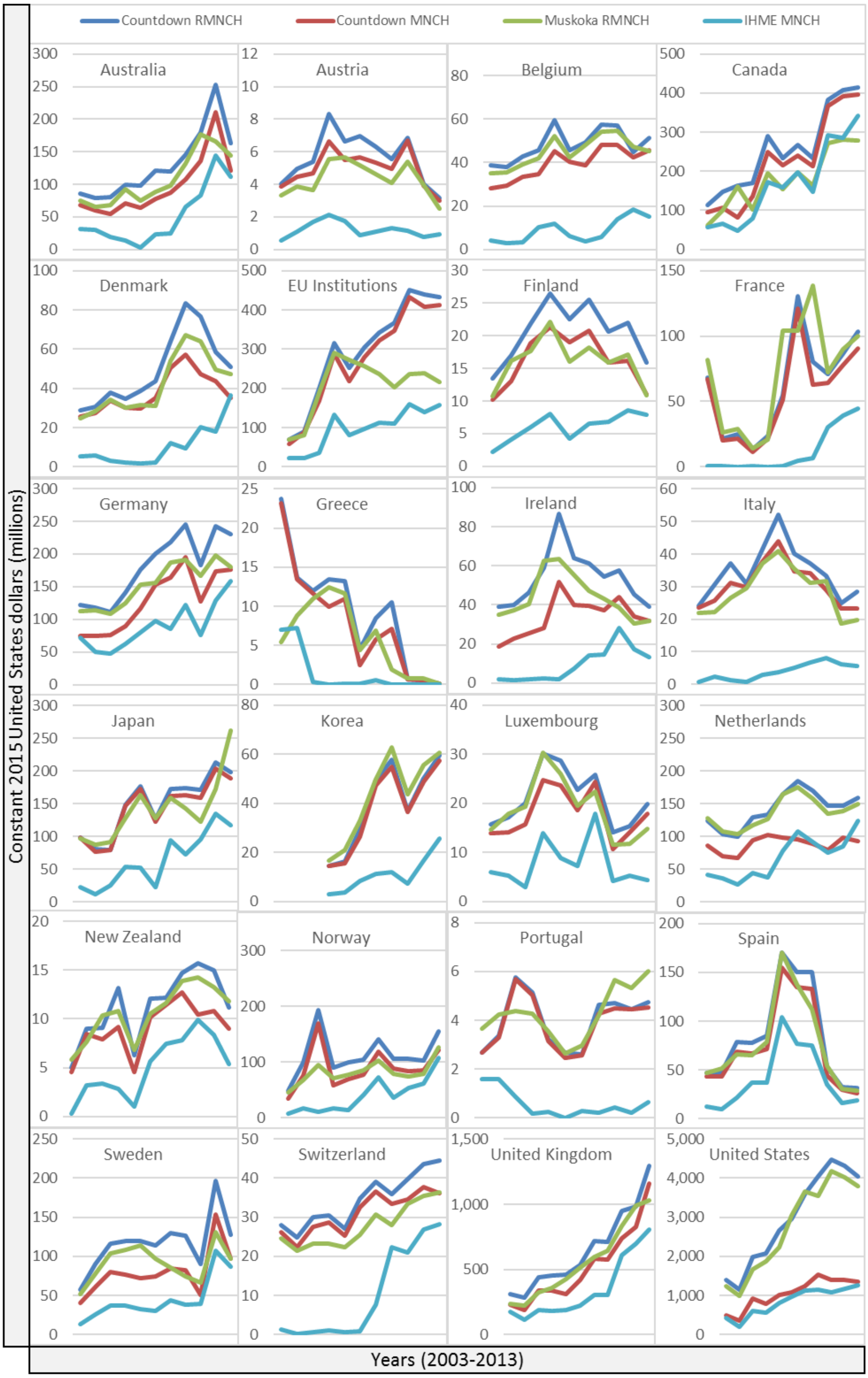

Supplementary Figure S12 Effects of differing RMNCH definitions and allocation procedures on estimates and trends in aid for RMNCH from 24 bilateral donors by recipient country, 2003-2013

This figure reflects our recreation of the Muskoka and IHME methods for identifying aid for RMNCH within the Countdown dataset, restricted to flows from 24 bilateral donors and disaggregated by recipient country. Flows to regional and unspecified recipients are not included. We held all other technical choices constant to isolate the effects on estimates of each method's RMNCH definitions and allocation procedures alone. IHME: Institute for Health Metrics and Evaluation. OECD: Organization for Economic Co-operation and Development.

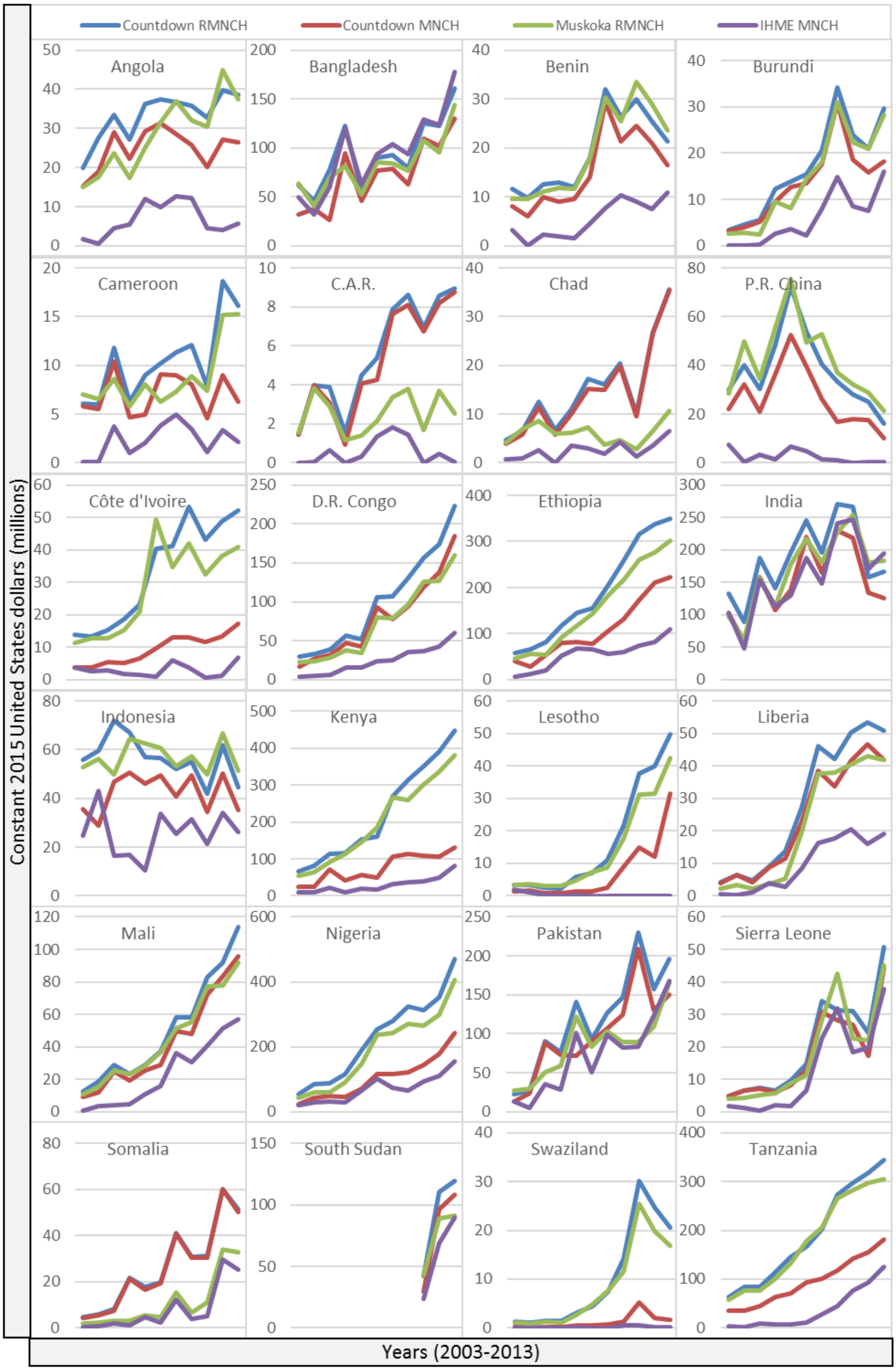

Supplementary Figure S13 Effects of different RMNCH definitions and allocation procedures on the distribution of aid for RMNCH per child from 24 bilateral donors in 2013

This figure reflects our recreation of the Muskoka and IHME methods for identifying aid to RMNCH within the Countdown dataset, restricted to flows from 24 bilateral donors. We held all other technical choices constant to isolate the effects on estimates of each method's RMNCH definitions and allocation procedures alone. Estimates were disaggregated by recipient country and flows to regional and unspecified recipients were excluded. Estimates were converted to a per-capita basis by dividing by the population of children in each country under the age of five. IHME: Institute for Health Metrics and Evaluation. OECD: Organization for Economic Co-operation and Development.

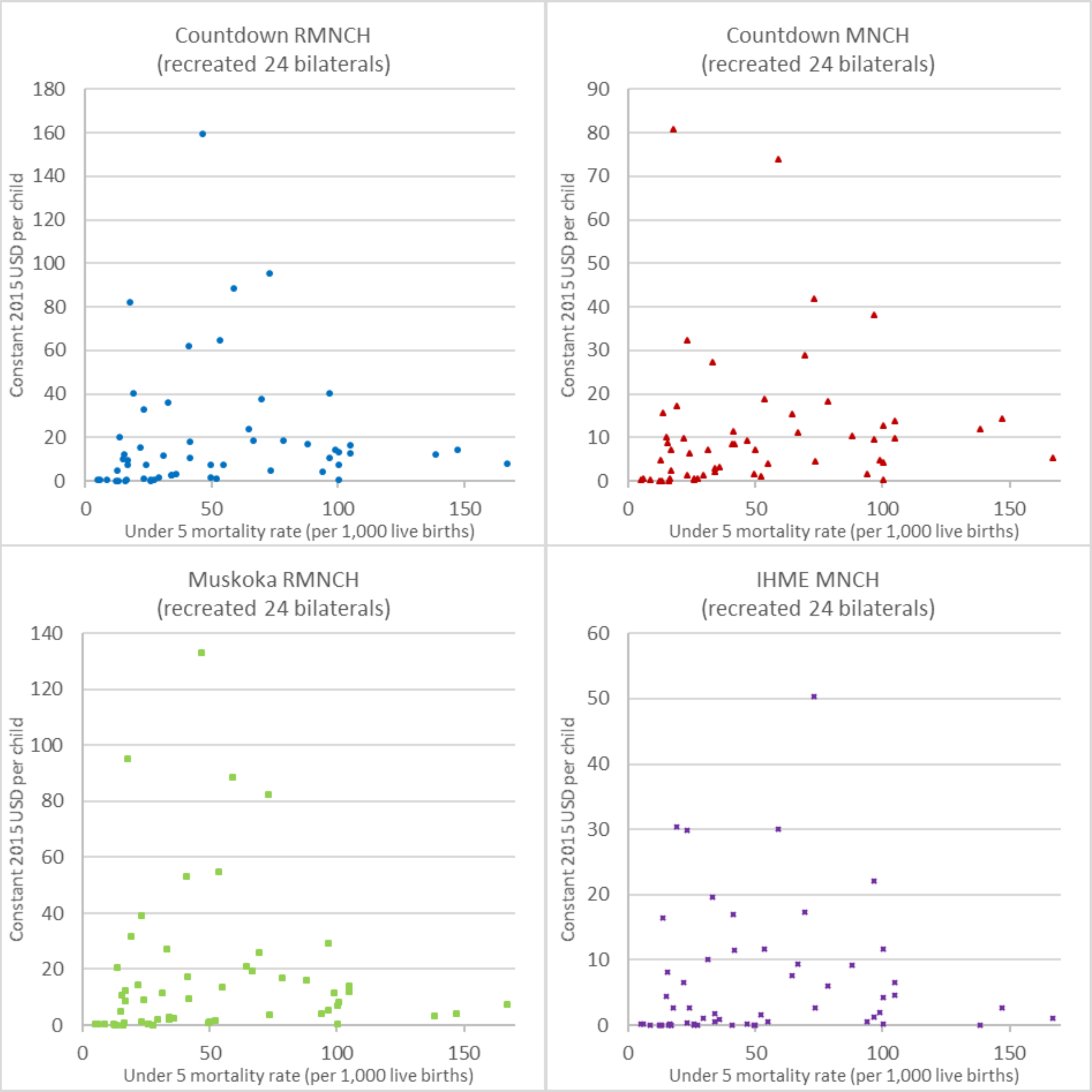

References

1. Tufte ER. The visual display of quantitative information. Cheshire, Conn.: Graphics Press; 1983.
2. United Nations. World Population Prospects: The 2017 Revision Population Database. 2017. https://esa.un.org/unpd/wpp/DataQuery/ (accessed 17 July 2017).
3. World Bank. World Development Indicators 2016. December 2015 ed. Washington, D.C.; 2016. http://databank.worldbank.org/data/reports.aspx?source=world-development-indicators
4. Institute for Health Metrics and Evaluation (IHME). Development Assistance for Health Database 1990-2016. Seattle, United States; 2017. http://ghdx.healthdata.org/record/development-assistance-health-database-1990-2016
5. Grollman C, Arregoces L, Martinez-Alvarez M, et al. Developing a dataset to track aid for reproductive, maternal, newborn and child health, 2003–2013. *Scientific Data* 2017; **4**: 170038. https://www.nature.com/articles/sdata201738
6. G8. Methodology for Calculating Baselines and Commitments: G8 member Spending on Maternal, Newborn and Child Health. 2010. http://www.g8.utoronto.ca/summit/2010muskoka/methodology.html
7. OECD. Converged Statistical Reporting Directives for the Creditor Reporting System (CRS) and the Annual DAC Questionnaire - Addendum 3: Reporting instructions for the RMNCH policy marker. Paris: OECD, 2014. https://www.oecd.org/dac/stats/documentupload/Addendum%203.pdf
8. USAID. 2015 VOLAG Report: Report of Voluntary Agencies Engaged in Overseas Relief and Development Registered with the U.S. Agency for International Development. 26 June 2018. https://www.usaid.gov/pvo/volag-report
9. OECD. Technical Guide to terms and data in the Creditor Reporting System (CRS) Aid Activities database. 2017. http://www.oecd.org/dac/stats/crsguide.htm (accessed 20 November 2017).
10. Institute for Health Metrics and Evaluation. Financing global health 2016: Development Assistance, Public and Private Health Spending for the Pursuit of Universal Health Coverage: Methods Annex. Seattle, WA: IHME, 2017. http://www.healthdata.org/policy-report/financing-global-health-2016-development-assistance-public-and-private-health-spending
